# Quantitative prediction of long-term molecular response in TKI-treated CML – Lessons from an imatinib versus dasatinib comparison

**DOI:** 10.1038/s41598-018-29923-4

**Published:** 2018-08-17

**Authors:** Ingmar Glauche, Matthias Kuhn, Christoph Baldow, Philipp Schulze, Tino Rothe, Hendrik Liebscher, Amit Roy, Xiaoning Wang, Ingo Roeder

**Affiliations:** 1 Institute for Medical Informatics and Biometry, Faculty of Medicine Carl Gustav Carus, TU Dresden, Dresden, Germany; 2 Bristol-Myers Squibb, Lawrenceville, NJ USA; 3 National Center for Tumor Diseases (NCT) partner site Dresden, Dresden, Germany

## Abstract

Longitudinal monitoring of BCR-ABL transcript levels in peripheral blood of CML patients treated with tyrosine kinase inhibitors (TKI) revealed a typical biphasic response. Although second generation TKIs like dasatinib proved more efficient in achieving molecular remission compared to first generation TKI imatinib, it is unclear how individual responses differ between the drugs and whether mechanisms of drug action can be deduced from the dynamic data. We use time courses from the DASISION trial to address statistical differences in the dynamic response between first line imatinib vs. dasatinib treatment cohorts and we analyze differences between the cohorts by fitting an established mathematical model of functional CML treatment to individual time courses. On average, dasatinib-treated patients show a steeper initial response, while the long-term response only marginally differed between the treatments. Supplementing each patient time course with a corresponding confidence region, we illustrate the consequences of the uncertainty estimate for the underlying mechanisms of CML remission. Our model suggests that the observed BCR-ABL dynamics may result from different, underlying stem cell dynamics. These results illustrate that the perception and description of CML treatment response as a dynamic process on the level of individual patients is a prerequisite for reliable patient-specific response predictions and treatment optimizations.

## Introduction

Chronic myeloid leukemia (CML) is a disease characterized by the expression of the BCR-ABL fusion protein in virtually all malignant cells in the vast majority of patients^1^. The affected leukemic stem cells have a competitive advantage over normal cells, leading to an initially slow but sustained expansion of the leukemic cell population. Untreated, the primary chronic phase (CP) of the disease eventually transforms into an accelerated phase followed by an acute blast crisis (BC), in which differentiation of functional blood cells is impaired and, therefore, physiological blood function is severely constrained, leading to the patient’s death if left untreated.

It is the molecular specificity of the BCR-ABL fusion gene that forms the basis of a highly efficient, targeted therapy by tyrosine kinase inhibitors (TKI). Already the introduction of the first-generation TKI imatinib significantly improved the treatment prognosis and increased five-year survival levels above 95%^2^. The availability of second-generation (dasatinib, nilotinib) and third-generation TKIs (e.g. bosutinib, ponatinib) further increased therapeutic options, including the treatment of a broad spectrum of secondary TKI resistant mutations^3^. Based on the success of TKIs, CML has developed into a showcase example for an efficient, targeted tumor therapy.

Although efficient treatment options are available, predicting the treatment success for a particular patient is still a challenge. In clinical practice, prognostic scores like the EUTOS, Sokal or Euro scores are commonly used to estimate a patient’s early response (e.g. achieving complete cytogenetic remission at 18 months) to front line TKI therapy^4^. Similarly, the molecular response at specific landmarks, such as three or six months of therapy, is measured in the peripheral blood using quantitative reverse transcriptase polymerase chain reaction (qRT-PCR) and used to distinguish treatment responders from non-responders^5,6^. Alternatively, a more dynamic perspective on therapy responses is taken by scores that address the “velocity of leukemia eradication”, such as ratios in the levels at two time points^7^ or the *halving time* of the tumor load^8–10^. Although these measures can be used to predict the average response probability of the particular treatment, none of them addresses the individual molecular long-term response in terms of its dynamic appearance or the individual risk of a late molecular relapse.

Considering BCR-ABL *dynamics* instead of using fixed time points for molecular response evaluation is a further alternative to describe treatment efficiency. Longitudinal qRT-PCR based monitoring of molecular response revealed that in most patients TKI treatment induces a biphasic decline of BCR-ABL transcript levels, which can be characterized by an initially steep decline, followed by a secondary moderate decline. While the first decline may result from the rapid depletion of actively cycling BCR-ABL positive leukemic cells, the second decline most likely represents the slow elimination of quiescent residual leukemic stem cells (LSC), which are less susceptible to cell kill due to their comparatively low cell cycle activity^11,12^. Following this line of argument, we support a dynamic description of the BCR-ABL response to characterize and predict long-term disease and treatment dynamics in individual patient, as previously suggested^11–14^.

Although the use of BCR-ABL dynamics in principle allows for a detailed characterization of the molecular response of individual patients, it is based on measurements obtained from the peripheral blood. Therefore, it has at least no direct implication for residual disease levels in the stem cell compartment within the bone marrow, which is clinically hard to assess. For that reason, we combine the statistical with a mechanistic model. This strategy allows us to statistically estimate patient-specific residual disease levels in the peripheral blood, even after the PCR monitoring has fallen below its quantification limit. At the same time, it enables us to estimate the treatment dynamics at the level of stem cells within the bone marrow, by describing the mechanisms of (stem) cell proliferation, differentiation and TKI effects. Specifically, our mechanistic modeling approach^11,14,15^ assumes two effects of the TKI therapy. First, it induces a BCR-ABL specific cytotoxic effect, while, secondly, it reduces the proliferative activity of leukemic stem cells. These assumptions are supported by clinical and experimental findings^16–19^. Obviously, the long-term follow-up of TKI treatment^20^ and clinical stop trials^21–23^ support the notion of a persisting pool of residual stem cells that rarely proliferate and only occasionally contribute to the peripheral blood at a minor or even undetectable level. We argue that under TKI treatment the pool of residual leukemic stem cells still undergoes a steady, albeit slow reduction in size. However, even after a long treatment time, a (dormant) reservoir may remain, which could lead to molecular relapses.

Here, we analyze response dynamics of 519 CML patients in chronic phase (CP) treated within the DASISION study, a randomized controlled clinical trial comparing first-line imatinib with dasatinib treatment^24^. Beside a rigorous statistical comparison of the molecular response dynamics, we describe an efficient method for applying our mechanistic mathematical model of TKI-treated CML to describe individual patient dynamics including appropriate confidence regions. Consequently, robust predictions for the molecular long-term response of imatinib- and dasatinib-treated patients are derived.

## Methods

### Clinical Data

The DASISION study (CA180-056, NCT00481247) is a multi-center, randomized trial to investigate the response of treatment-naïve CP-CML patients (N = 519) medicated daily with either imatinib (median dose was 400 mg with a range of 125 to 741 mg) or dasatinib (median dose was 99 mg with a range of 21 to 139 mg)^25^. The primary endpoint was the confirmed complete cytogenetic remission (cCCyR) by 12 months. Secondary endpoints included rates of CCyR and MMR, times to CCyR and MMR, as well as progression free and overall survival. The data set available for statistical analysis and modeling is based on the minimum follow-up of 5 years, analyzed for 259 dasatinib- and 260 imatinib-treated patients. The molecular response was assessed by reporting BCR-ABL transcript levels obtained from real-time quantitative polymerase chain reaction (RQ-PCR). The assay was performed in a centralized lab and data is normalized to the international scale (IS) with ABL being the reference gene. Molecular analysis was performed at baseline (month 0) and then every three months after treatment was initiated until month 24. Thereafter, the molecular assessment was conducted at least every 6 months until the end of study or disease progression.

**Figure 1:**
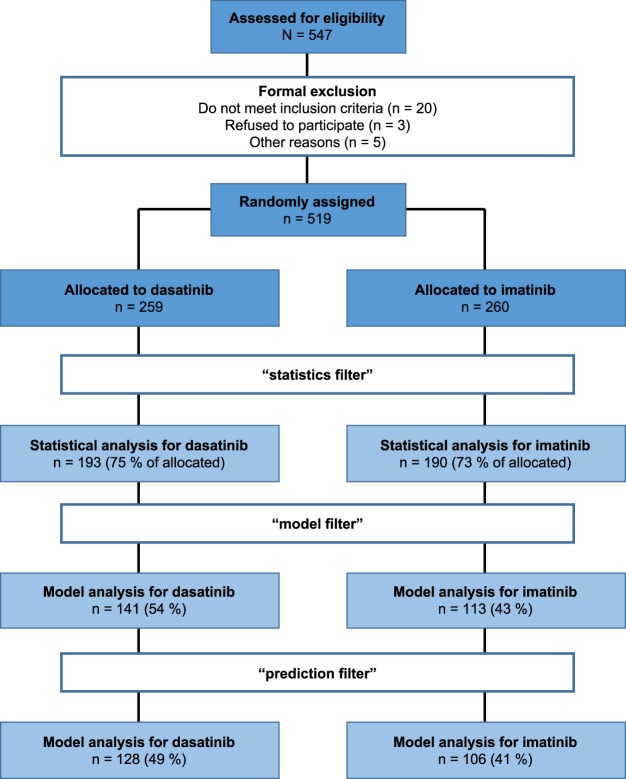
Data flowchart. The flowchart illustrates the selection process of patient data for statistical and model analyses.

In order to reliably estimate dynamic parameters of the treatment response (i.e. BCR-ABL decline slopes), we restricted our analysis to those patients that conform to the following criteria, denoted as “*statistics filter*”: (1) minimum of 5 time points evaluated, (2) at least two BCR-ABL/ABL measurements within first 4.5 months (to ensure estimation of the initial slope), (3) first BCR-ABL/ABL measurements prior or within first 15 days after treatment start (to ensure an estimate of the initial tumor load), (4) minimal follow up at least 18 months (to ensure a reliable estimation of the secondary slope), (5) no periods larger than 24 months without available BCR-ABL/ABL measurements (to exclude rarely monitored patients). Applying this selection, a total of 383 patients (193 in dasatinib arm (75% of allocated), 190 in imatinib arm (73% of allocated)) were retained and used in the statistical analysis. As our mechanistic mathematical model (see below) is only able to describe dynamic responses with a bi-phasic decline, i.e. the initial BCR-ABL decline *α* is steeper compared to the second decline *β*, we applied a further selection strategy (“*model filter*”): (6) initial slope (*α*) > secondary slope (*β*) > 0 (a detailed explanation of the slopes is provided below). Finally, the quantification of the prediction accuracy is only realized for patients, which fulfill criterion (7) a complete 5 year follow up (“*prediction filter*”). A summary of the data analysis/modelling inclusion criteria along with the actually used patient numbers is provided in Fig. 1.

### Statistical analysis

**Figure 2:**
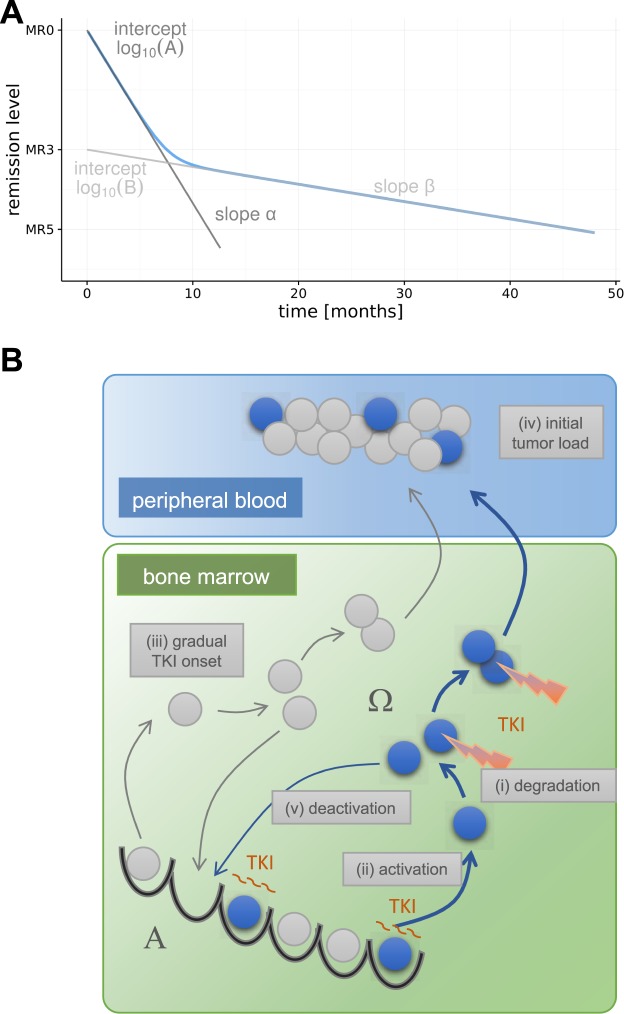
Statistical und mechanistic model. (**A**) Bi-exponential model for the description of time course data, parameterized by the intercepts A, B, and the initial slope α and the secondary slope β. (**B**) Model setup of the mechanistic, single-cell based clonal competition model of CML pathogenesis and treatment. Leukemic cells are shown in blue, normal cells in grey. Both cell types change between a state of proliferative inactivity (**A**) and a proliferative state (Ω) before cells differentiate into peripheral blood. TKI activity is indicated by the cytotoxic effect and the prolonged quiescence of leukemic stem cell in state A. The five parameters modified for the simulation screen are identified by roman numerals (i)–(v).

To quantify a patient’s treatment response, we use the corresponding BCR-ABL transcript levels, described by the logarithm of the relative abundance (in %) of the BCR-ABL transcript, i.e. LRATIO = log_10_(BCR-ABL/ABL * 100). The nonlinear relationship between the time under treatment (t) and the LRATIO per patient are modelled by the logarithm of a bi-exponential function, i.e.: LRATIO(t) = log_10_ (A^−αt^ + B^−βt^), with parameters A, B, *α*, and *β* and the convention that A > B > 0 to make the parameters unambiguous. The bi-exponential function on the log-scale resembles a smoothed version of a piecewise linear function with a single break point (cf.^14^). The parameters A and B can be interpreted as the intercepts of the two approximating line segments, while *α* and *β* represents the slopes of these lines (Fig. 2A). We constrain *α* > 0 to reflect the expected initial decline. The parameter β was not restricted (i.e. β $\in \,{\mathbb{R}}$), allowing for either a decrease or an increase in BCR-ABL levels in the second phase (cf.^12^).

For the assessment of treatment differences between imatinib and dasatinib therapy, we apply a population-based non-linear mixed effect (NLME) model using a maximum likelihood (ML) estimation. The model selection, i.e. the decision which terms to include in the final NLME-model, is guided by Akaike information criterion (AIC). We apply Wald tests to assess statistical significance of model parameters. To fit the bi-exponential function to the measurements for each individual patient *i*, we used the non-linear least squares method instead of the patient-level predictions of the NLME model, obtaining a fit that minimizes the residual squared errors for each patient. In both routines (population- and patient-based), BCR-ABL values below the quantification limit (QL) of the RQ-PCR, are treated as (left-)censored values. I.e., we assume that the true BCR-ABL level is at least as low as the QL, but it could also be lower. We refer to the estimated parameter tuple as ${\hat{\rho }}_{i}^{\tau }=(A,\,\alpha ,B,\,\beta )$ and to the residual variance as ${\hat{\sigma }}_{i}^{2}$. Confidence intervals for the mean LRATIO-value at a given time point are calculated via parametric bootstrap. For technical details see Supplementary Methods.

To fit and evaluate the NLME model, we applied the software “Monolix” (version 2018R1)^26^. All other statistical evaluations were done using the statistical programming environment R^27^. For further technical details, we refer to the Supplementary Methods.

### Mechanistic model

#### Model structure and implementation

CML is modeled as a clonal competition phenomenon between normal hematopoietic and leukemic (stem) cells (Fig. 2B). This concept is implemented as a single cell-based model that was originally developed to describe murine hematopoiesis^28,29^. By upscaling the stem cell number and adapting cell turnover rates (see^11^ for details), the model has been successfully applied to the human situation, in particular to CML pathogenesis and treatment^11,14,15,30^. In order to explain the competitive advantage of untreated leukemic cells compared to normal HSCs, we assume an increased, unregulated proliferative activity of leukemic cells. The treatment of CML patients with TKI is assumed to induce a specific cytotoxic effect and an inhibition of the proliferative activity of leukemic stem cells (Fig. 2B, indicated in red). Technically, the cytotoxic effect is modeled by a selective kill of a fixed percentage of TKI affected leukemic cells per time step (degradation rate r_deg_), while the proliferation inhibition is modeled by a reduction of the activation of leukemic cells into cycle (reduced activation function ${{\rm{f}}}_{{\rm{\omega }}}^{{\rm{CML}}}$). In order to adapt our model to the initial patient’s response during the first six months of treatment we assume a gradual onset of the effect of TKI activity. Technically, this is implemented as a rate (r_trans_) describing how fast LSCs are affected by the TKI (i.e, transition rate from a naïve to a TKI-affected state). We do not assume a mechanistic difference between the effect of imatinib and dasatinib in the model, but we consider the patient-specific treatment response as the result of an individual tumor growth rate and an individual TKI response, which might quantitatively differ between patients and/or their treatment scenario. The tumor load in the peripheral blood, which is clinically measured in terms of BCR-ABL transcript levels, is approximated in the mathematical model by the proportion of leukemic (i.e. BCR-ABL positive) cells in the population of fully differentiated cells according to the following equation:

$${\rm{BCR}} \mbox{-} {\rm{ABL}}/{\rm{ABL}}\,\approx \,({n}_{1}/({n}_{1}+2{n}_{2}))\cdot 100 \% .$$

Herein, *n*_1_ denotes the number of leukemic cells and *n*_2_ the number of normal cells^31^. Further details of the model implementation are provided in the Supplementary Methods.

#### Parameter estimation

To derive a consistent model fit for an individual patient, we previously used the same mechanistic model^14^. However, the optimization procedure comprised the variation of two parameters only, namely (i) the patient specific degradation rate r_deg_ and (ii) the activation function ${{\rm{f}}}_{{\rm{\omega }}}^{{\rm{CML}}}$. Meanwhile, it became evident that also the influence of further treatment- and patient-specific parameters cannot be neglected. Beyond (iii) the rate of gradual TKI onset (r_trans_), we identified  (iv) the initial tumor load at therapy start (described by the LRATIO at time point t = 0, LRATIO_init_) and (v) the rate by which leukemic stem cells re-enter into quiescence (deactivation, ${{\rm{f}}}_{{\rm{\alpha }}}^{{\rm{CML}}}$) to be additional sensitive determinants of the response dynamics (illustrated in Fig. 2B). For reasons of efficiency, we realized a systematic, large-scale screen of a defined parameter space and deposited the results in a *look up table*. In brief, for each of the five parameters (i–v), we determined a plausible range, subdivided the corresponding intervals and assigned 10 (LRATIO_init_), 16 (rdegr_deg_), 13 (r_trans_), 10 (${{\rm{f}}}_{{\rm{\omega }}}^{{\rm{CML}}}$) and 13 (${{\rm{f}}}_{{\rm{\alpha }}}^{{\rm{CML}}}$) values at which we evaluated the model in all combinations. This resulted in the set Θ of 270,400 different parameter configurations ${\vartheta }=({{\rm{f}}}_{{\rm{\omega }}}^{{\rm{CML}}},{\,f}_{{\rm{\alpha }}}^{{\rm{CML}}},{{\rm{r}}}_{{\rm{\deg }}},{{\rm{LRATIO}}}_{{\rm{init}}},{{\rm{r}}}_{{\rm{trans}}})$.

In order to determine the set ${\hat{{\rm{\Theta }}}}_{i}^{\tau }$ of suitable parameter configurations of the mechanistic model that fit the time course of BCR-ABL/ABL levels for patient *i*, we apply the following strategy: (1) We derive the bi-exponential fit ${\hat{\rho }}_{i}^{\tau }$ according to an optimization routine (see Supplementary Methods) for the clinically available BCR-ABL levels up to time *τ*. Furthermore, we provide corresponding point-wise 95%-confidence intervals for the fit $\,{\hat{\rho }}_{i}^{\tau }$. (2) Using the above described look-up table, we identify all parameter configurations of the mechanistic model, for which the bi-exponential fit of the model-predicted BCR-ABL time courses are completely contained within the statistically determined confidence region (mechanistic model fitting). These identified tuples constitute the set of suitable parameter configurations ${\hat{{\rm{\Theta }}}}_{i}^{\tau }$. We order the simulated BCR-ABL dynamics in ${\hat{{\rm{\Theta }}}}_{i}^{\tau }\,\,$according to their distance (similarity) to the bi-exponential fit of the data ${\hat{\rho }}_{i}^{\tau }$ and refer to ${\hat{\vartheta }}_{i}^{\tau }$ *as* the optimal (best-fitting) parameter configuration in ${\hat{{\rm{\Theta }}}}_{i}^{\tau }$. Further technical details are provided in the Supplementary Methods.

#### Cross validation for the prediction accuracy

In order to quantify the prediction accuracy of our mechanistic model, we compare the set of suitable parameter configurations from “reduced” data sets, i.e. for a 2, 3 or 4 year BCR-ABL monitoring (${\hat{{\rm{\Theta }}}}_{i}^{2y}$, ${\hat{{\rm{\Theta }}}}_{i}^{3y}$, ${\hat{{\rm{\Theta }}}}_{i}^{4y}$), respectively, with the suitable parameter configurations for the complete 5-year follow-up ${\hat{{\rm{\Theta }}}}_{i}^{5y}$. Specifically, we determine the overlap of 5-year predictions for BCR-ABL levels based on ${\hat{{\rm{\Theta }}}}_{i}^{5y}\,\,$with predictions based on the reduced observation periods ${\hat{{\rm{\Theta }}}}_{i}^{ < 5y}$. Now, we categorize the predictions by evaluating the overlap of the predicted confidence intervals at the end of the 5-year period (Supplementary Fig. S2). We denote 5-year predictions as *true positives* (TP) that are within the confidence intervals at 5 years determined by both the reduced as well as the complete data set. *True negatives* (TN) are defined as predictions being neither within the confidence interval at 5 years derived from the reduced nor the complete data set. *False positives* (FP) are predictions that are within the confidence interval determined by the reduced data set, but not within the confidence interval at 5 years determined by the complete data set. *False negatives* (FN) refer to predictions that are not within the confidence intervals derived from the reduced data set but within the confidence interval determined by the complete data set.

### Availability of data and material

Data are available from the authors upon reasonable request and with permission of Bristol-Myers Squibb.

## Results

### Comparing average treatment response of dasatinib vs. imatinib

**Figure 3:**
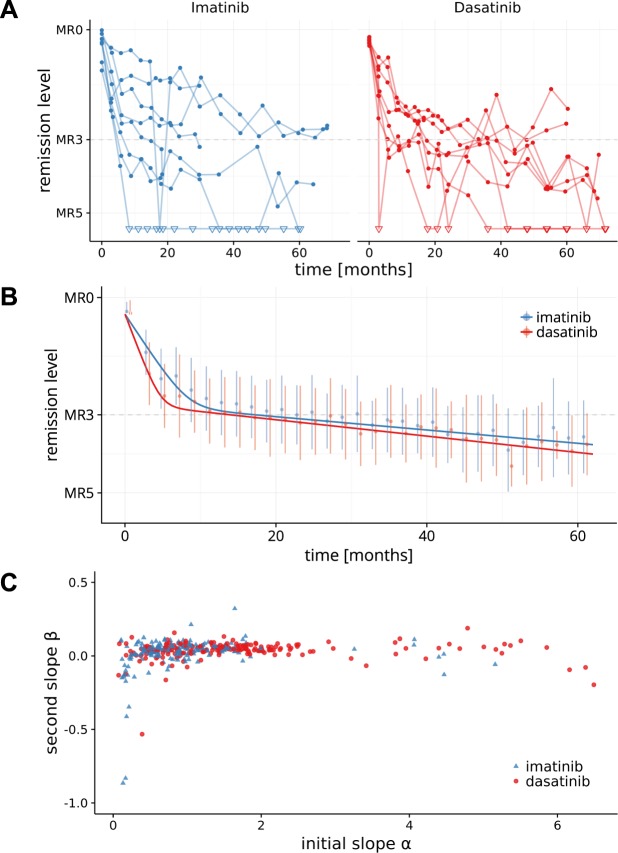
Dynamics of treatment response. (**A**) Time course data of a random subset of 8 patients per treatment cohort. BCR-ABL levels below detection threshold are indicated by open triangles. (**B**) Time-course of mean BCR-ABL levels (±SD) are shown for intervals of 2 months. The lines correspond to the fixed-effect predictions of the mean for the imatinib and the dasatinib cohorts. (**C**) Scatter plot illustrating the missing correlation between initial slope α, and secondary slope β for all available, individually fitted patient time courses (Spearman correlation [95% confidence interval]: imatinib r = 0.27 [0.13; 0.40], dasatinib: r = 0.17 [0.03; 0.31], all: r = 0.24 [0.14; 0.33]).

Individual treatment responses in both arms of the DASISION trial show a considerable degree of heterogeneity between individual patients (Fig. 3A, Supplementary Fig. S3). However, the typical bi-phasic BCR-ABL dynamic is observed in the majority of patients. This also holds true for a mean time course of the patients in the two treatment arms (Fig. 3B). Although the molecular response dynamics for imatinib and dasatinib are qualitatively similar, there are quantitative differences. We demonstrate statistically significant effects in the average response dynamics (Fig. 3B, Table 1). Specifically, dasatinib treatment yields a *faster* initial reduction of BCR-ABL levels (i.e. significantly larger slope parameter *α*, p < 0.0001). Although, we also observed a difference in the average long-term BCR-ABL decline between the two treatments (p = 0.0323), the dasatinib-induced β-slope is only marginally steeper compared to imatinib. We did neither detect a statistically significant difference in the initial intercept parameter A, which approximates the initial BCR-ABL levels (p = 0.909) nor in the second intercept parameter B (p = 0.361). Our findings confirm recently reported results^24^ that, on average, a major molecular response (i.e. BCR-ABL levels <0.1% = MR3) is achieved earlier in the dasatinib arm (after 22 months) compared to the imatinib arm (after 36 months). These results are robust with respect to uncertainties for BCR-ABL values >10%, potentially induced by the use of the ABL reference gene (see Supplement material).

**Table 1:** Fixed-effect parameter estimates from the selected non-linear mixed effect model (NLME).

Parameter	Imatinib	Dasatinib	p-value
A	37.375		n.a.
α	0.674	1.168	<0.0001
B	0.196		n.a.
β	0.039	0.048	0.0323

Treatment differences are considered for slope parameters α and β. p-values for the treatment effect on the bi-exponential parameters are based on Wald tests for equality of the two groups. n.a. = not applicable.

### Quantification and prediction of individual treatment responses

Our results show an accelerated, more pronounced average initial molecular response in the dasatinib-treated cohort compared to the imatinib arm (Fig. 3B). However, on the level of individual patients, there is considerable heterogeneity in the BCR-ABL decline dynamics (both, initially and long-term) that exceeds the differences in the mean response between the two cohorts (c.f. Fig. 3A, Supplementary Fig. S3). The patients’ heterogeneity is not only reflected in the BCR-ABL dynamics, it also applies to other prognostic scores or landmarks, such as MR level at 3 or 6 month^5,6^ or the halving time^8–10^. Here, we confirm a close correspondence of halving time and initial BCR-ABL decline α with respect to their prognostic value in achieving major or deep molecular response, with a steeper α-slope /shorter halving time being related to a better average response (Supplementary Fig. S1). In fact, one can show that the halving time is a surrogate measure of the slope of the initial BCR-ABL decline (α) and, thereby, closely resembles its predictive value. At the level of individual patients, however, neither a short halving time nor a steeper initial BCR-ABL decline guarantees a favorable long-term outcome. The lacking prognostic power of the initial treatment response on the individual long-term behavior is also supported by the fact that there is no relevant correlation between the initial (*α*) and the secondary (*β*) slope of the BCR-ABL dynamics (Fig. 3C, Spearman correlation coefficient: r = 0.24). With respect to the treatment failure, also neither the halving time (Supplementary Fig. S1) nor the initial slope *α* (data not shown), are consistently predictive. In contrast, the secondary *β* slope allows to identify a subpopulation of patients with a higher rate of progression events both for the dasatinib and the imatinib arm (Supplementary Fig. S1).

Taken together, these results show, that an individual, patient-specific prognosis of long-term treatment efficiency is not possible on the basis of early treatment characteristics alone. Therefore, we suggest to combine information from early and late treatment dynamics to estimate the patient’s individual response characteristics. This strategy, however, would not work in case of incomplete information, e.g. due to short molecular monitoring periods or due to undetectable BCR-ABL levels, which hinder the direct estimation of response dynamics. Furthermore, although the BCR-ABL levels in the peripheral blood partially reflect the residual disease levels of stem cells, considerable information is lacking to estimate the patient’s long-term response. To overcome these insufficiencies, we suggest to combine the statistical description (i.e., the estimation of α and β slopes) with a mechanistic modelling approach, which allows to infer the latent stem cell dynamics from the molecular monitoring in the peripheral blood and, therefore, to provide better predictions of the individual molecular long-term treatment response.

### Mechanistic modelling of individual treatment responses

As motivated above, we complement the statistical modelling by the application of a mechanistic model of TKI-treated CML patients^11,15^. In order to estimate patient-specific parameters of the mechanistic model, we previously suggested to use a nonlinear regression model that patient-wise relates the estimated initial and long-term slope of the corresponding clinically observed BCR-ABL dynamic to a particular value of the two most critical mechanistic model parameters, namely the degradation rate r_deg_ and the reduced activation function ${{\rm{f}}}_{{\rm{\omega }}}^{{\rm{CML}}}$. Although the model provided suitable approximations, the adaptation failed in some scenarios^14^. This approach has been considerably improved, such that we are now able to provide a consistent model description for *all* of the considered patients in the IRIS, CML-IV and DASISION trials. To achieve this, we systematically varied five key model parameters and simulated BCR-ABL time courses for more than 270,000 different parameter combinations $\,\vartheta$, i.e., generating a large population of “virtual patients” (for technical details see Material & Methods and Supplementary Methods). The results of this systematic, large-scale parameter screen have been stored in a look-up table and can be used to efficiently identify parameter configurations that optimally represent a given, i.e. clinically observed, patient-specific BCR-ABL dynamic.

**Figure 4:**
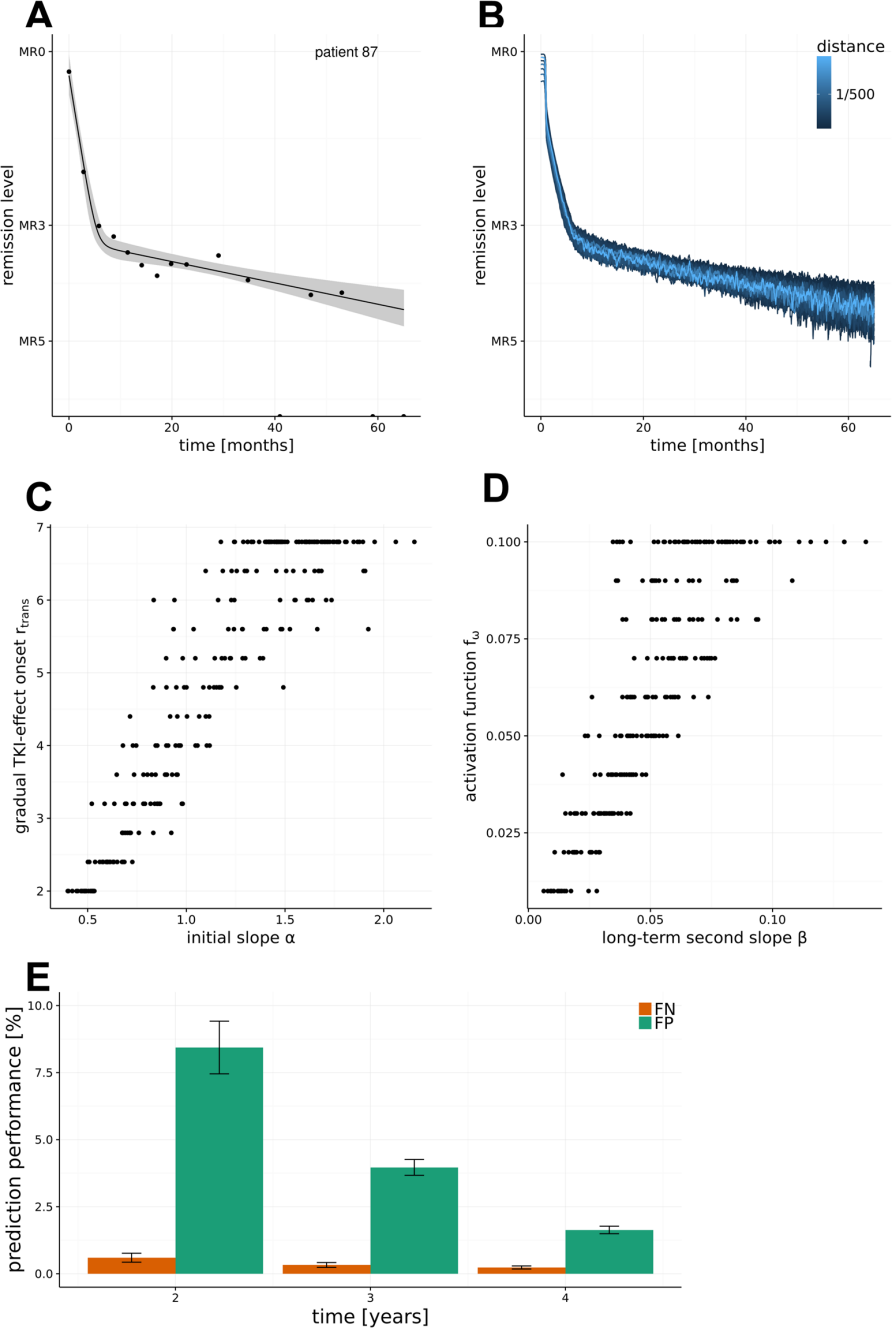
Estimation of model parameters and prediction accuracy. (**A**) The optimal fit of the bi-exponential regression model $\,{\hat{\rho }}_{i}^{\tau }$ is shown along with a point-wise confidence interval for one patient *i*. (**B**) Identification of all parameter configurations $\,{\hat{{\rm{\Theta }}}}_{i}^{\tau }$, for which the bi-exponential fit of the resulting model simulation is contained within the confidence region of the patient’s kinetic. (**C**) Scatter plot relating the initial slope α of each patient’s response with the rate of gradual TKI-effect onset (r_trans_) obtained for the most suitable model simulation ${\hat{\vartheta }}_{i}^{\tau }$. (**D**) Scatter plot relating the long-term decline β of each patient’s response with the specific activation rate of the residual LSC (${{\rm{f}}}_{{\rm{\omega }}}^{{\rm{CML}}})$ obtained for the most suitable model simulation ${\hat{\vartheta }}_{i}^{\tau }$. (**E**) False positives (FP) and false negatives (FN) rates for predictions of 5 year outcomes as a function of shorter observation periods (n = 234).

Figure 4A shows the clinically determined BCR-ABL levels of a particular patient *i* over an observation period of almost 5 years alongside with the estimated bi-exponential regression model ${\hat{\rho }}_{i}^{5y}$ and point-wise confidence intervals. Using the above described look-up table, we identified all suitable parameter configurations of the mechanistic model ${\,\hat{{\rm{\Theta }}}}_{i}^{5y}$, which generate simulated BCR-ABL dynamics that fall within the estimated confidence region (Fig. 4B). Model fits of other DASISION patients are provided in Supplementary Fig. S5. In general, the number of parameter configurations within ${\hat{{\rm{\Theta }}}}_{i}^{\tau }$ scales with the residual variance ${\hat{\sigma }}_{i}^{2}$ of the bi-exponential data fit $\,{\hat{\rho }}_{i}^{\tau }$. In other words, a patient dynamic that closely adheres to a biphasic decline characteristic leads to a narrower confidence band, which contains fewer suitable parameter configurations. In contrast, fluctuating and/or few data points lead to a fit with a broader confidence region. A broader confidence region, consequently, usually leads to more suitable mechanistic model simulations. This results in greater uncertainty and hampers the identification of a unique model explanation.

Comparing the parameters of the best-fitting mechanistic model ${\vartheta }_{i}^{\tau }$ with the corresponding statistical parameters ${\hat{\rho }}_{i}^{\tau }$, we observe a positive correlation between the rate of the gradual TKI-effect onset (r_trans_) and the slope of the initial decline *α* (Fig. 4C). I.e., the model implies that a faster drug action results in an accelerated reduction of the initial tumor burden. Furthermore, the cell proliferation activation function (${{\rm{f}}}_{{\rm{\omega }}}^{{\rm{CML}}})\,\,$correlates with the long-term decline (slope *β*). This suggests that the dynamics of the long-term response are predominantly determined by the rate of residual LSC activation (Fig. 4D). Because the gradual TKI-effect onset (described by r_trans_) is only relevant for early time points, our result can be interpreted as follows: The overall TKI-induced kill of BCR-ABL positive cells consists of two sub-effects, a background kill rate (described by model parameter r_deg_), which acts over the entire administration period of a TKI, and an additional effect, which is only acting shortly after onset of treatment (described in the model by the combination of r_deg_and gradual TKI-effect onset r_trans_). Comparing the parameters for the optimal model fits ${\hat{\vartheta }}_{i}^{\tau }$ for all patients, one observes a tendency towards higher rates of gradual TKI-effect onset (r_trans_) in the dasatinib cohort, reflecting an accelerated drug response (Supplementary Fig. S3). Only minor differences of other model parameters are observed for imatinib- vs. dasatinib-treated patients.

### Model prediction for long-term treatment dynamics

As introduced above, the set of suitable parameter configurations ${\hat{{\rm{\Theta }}}}_{i}^{\tau }$ consistently describes the observed treatment dynamics of patient *i*. However, to support clinical decision-making, the model should additionally yield valid predictions for yet unobserved data, e.g. expected BCR-ABL values beyond the current treatment period *τ*. To test the quality of model-predicted BCR-ABL dynamics based on available clinical data, we compared the predictions ${\hat{{\rm{\Theta }}}}_{i}^{2y}$, ${\hat{{\rm{\Theta }}}}_{i}^{3y}$, ${\hat{{\rm{\Theta }}}}_{i}^{4y}$ derived on the basis of a (reduced) 2, 3 and 4 year data follow-up, with the complete 5 year follow-up ${\hat{{\rm{\Theta }}}}_{i}^{5y}$ for each eligible patient (Supplementary Fig. S2, see also Material and Methods, Supplementary Methods). Figure 4E illustrates that both, the false negative rate (FN) and the false positive rate (FP) decline with longer follow up. Consequently, the fraction of correct predictions (i.e. TP + TN) increases. Specifically, our data shows that already predictions derived on the basis of 2-year BCR-ABL monitoring are compatible with the 5-year follow-up predictions in more than 90%, i.e. FP + FN < 10% (see Fig. 4E). To test for result robustness with respect to uncertainty of BCR-ABL/ABL ratios >10%, we repeated the analysis for the scenarios in which all these measurements were either down-weighted by a factor ½ or omitted from the analysis. No qualitative difference of the results was observed (Suppl. Figs S6 and S7).

We would like to point out that the number of suitable parameter sets ${\hat{{\rm{\Theta }}}}_{i}^{\tau }$ depends on the variability of the data: the broader the confidence region the more parameter configurations (“virtual patients”) are considered to be “consistent” with the data. Thus, “consistency” (in the sense used here) and identifiability of the true patient-specific parameter configuration are anti-correlated. We suggest to interpret the prediction accuracy (i.e., 1 - error rates) conditionally on the quality of the data. Meaning, for a particular interpretation, error rates should only be compared with a normalization according to the positive values.

### Estimating leukemic stem cell dynamics

**Figure 5:**
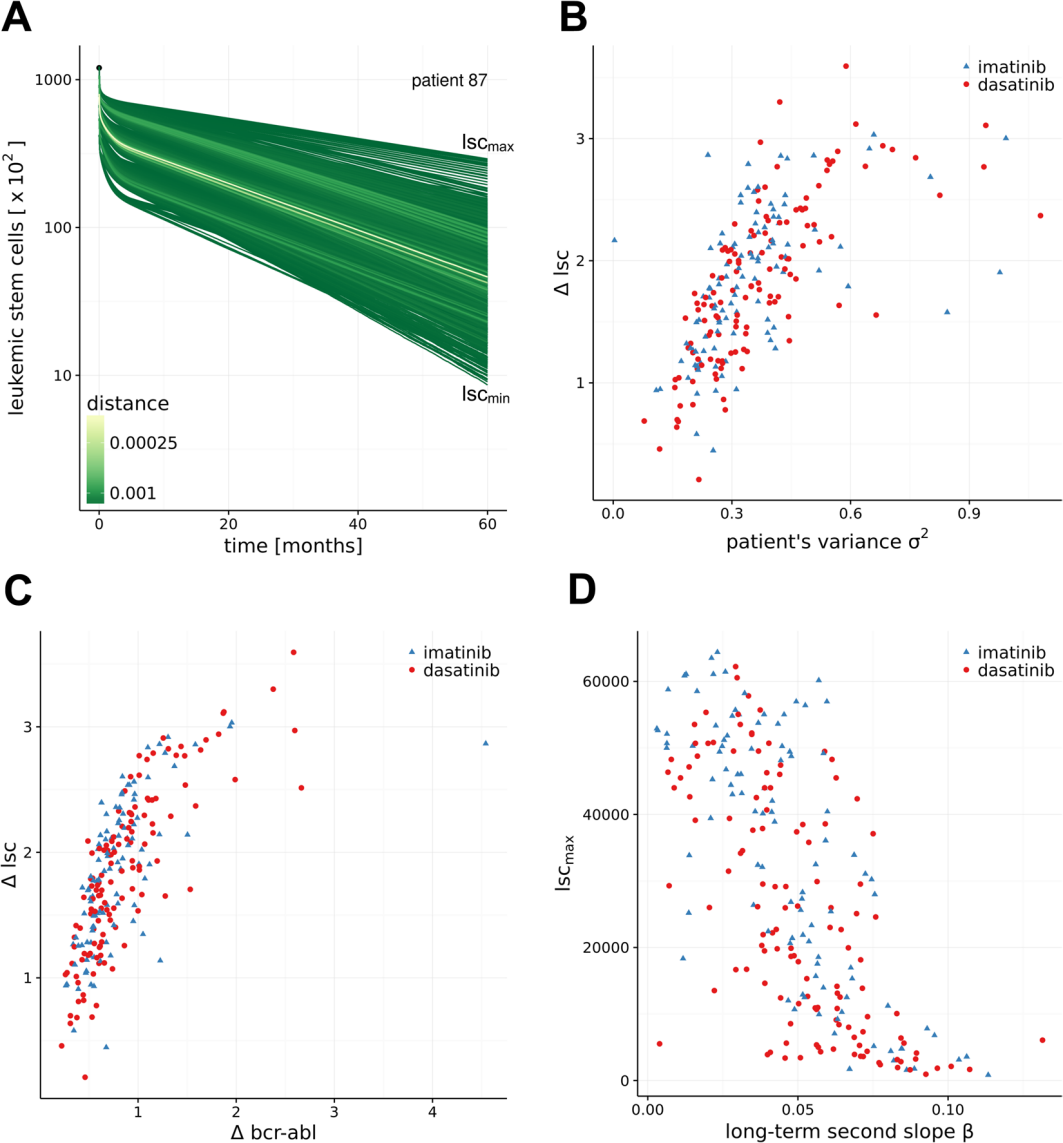
Estimating residual stem cell numbers. (**A**) Variability for predictions of residual leukemic stem cell (LSC) numbers for the model simulations ${\hat{{\rm{\Theta }}}}_{i}^{\tau }\,$in Fig. 4A,B. Every green line corresponds to one suitable parameter configuration within ${\hat{{\rm{\Theta }}}}_{i}^{\tau }\,$indicated in blue in Fig. 4B. The green coloring scheme indicates the distance of the simulation results to the bi-exponential approximation ${\hat{\rho }}_{i}^{\tau }\,$of patient *i*. lsc_max_ and lsc_min_ refer to the maximal and minimal number of predicted LSCs at 5 years. (**B**) Correlation of the residual variance ${\hat{\sigma }}_{i}^{2}$ of the model fit (as a measure of data quality) to the variability of the number of predicted LSCs. (**C**) Correlation of the width of the prediction interval in the peripheral blood and the variability of the number of predicted LSCs. (**D**) Correlation of the second slope β of the patient fit ${\hat{\rho }}_{i}^{\tau }\,$with the maximal number of predicted LSCs.

Beyond the prediction of BCR-ABL dynamics in the peripheral blood, a particular advantage of the mechanistic modelling is the ability to predict the underlying stem cell dynamics. Thus, the modelling approach in principle allows to derive estimates about the dynamics of leukemic (i.e. BCR-ABL positive) stem cells (Fig. 5A). In Fig. 4B we visualize the suitable parameter configurations ${\hat{{\rm{\Theta }}}}_{i}^{\tau }$ for an individual patient dynamic *i*. These PB BCR-ABL dynamics may, however, result from very different, underlying stem cell dynamics. As a simple example, a particular, slow decline of BCR-ABL levels in a given patient could be generated by a lower toxicity of the TKI or, alternatively, by a more pronounced quiescence of the leukemic stem cells. Our model analysis clearly shows that although the dynamics in the peripheral blood might appear very similar, the underlying stem cell dynamics can distinctly differ. Figure 5A shows one example of various stem cell dynamics which according to the mechanistic model all result into similar PB BCR-ABL dynamics (i.e. simulations within ${\hat{{\rm{\Theta }}}}_{i}^{\tau }$). Further examples are provided in the Supplement Fig. S4. These results demonstrate that even detailed information on the BCR-ABL dynamics in the PB is not always a reliable surrogate measure for the residual disease in terms of the remaining leukemic stem cells.

Analyzing the full patient cohort of the DASISION trial, we observed that the residual variance ${\hat{\sigma }}_{i}^{2}\,\,$of the model fit (i.e. the “quality” of the data) correlates with the width of the prediction interval for the residual leukemic stem cells $({\rm{\Delta }}\mathrm{lsc}={\mathrm{log}}_{10}({{\rm{lsc}}}_{{\rm{\max }}})-{\mathrm{log}}_{10}({{\rm{lsc}}}_{{\rm{\min }}}))$ at 5 years (Fig. 5B). Furthermore, the width of the predictions from the parameter configurations ${\hat{{\rm{\Theta }}}}_{i}^{\tau }$ in peripheral blood at 5 years (Δbcr-abl = log_10_ (bcr-abl_max_) − log_10_ (bcr-abl_min_)) only moderately correlates with the corresponding width of the stem cell level Δlsc (Fig. 5C). Reassuring the validity of our approach, we also observed that the maximal number of residual leukemic cells inversely correlates with the slope *β* characterizing the second decline (Fig. 5D). Thus, our model suggests that the steepness of the long-term decline of BCR-ABL levels in PB (i.e., *β* slope) is an indicator of the number of residual LSC. A steeper *β* decline leads to lower residual leukemic stem cell level (given a certain treatment duration).

Given our suggested approach, we are in principle able to derive prediction intervals for the number of residual leukemic stem cells at any point during TKI treatment, although the uncertainty of this predication is higher (larger confidence regions) than for the prediction of residual disease levels in the peripheral blood. To validate the model with respect to its prediction power of residual LSC numbers, it needs to be compared either to BCR-ABL levels in the stem cell(-enriched) population of bone marrow cells (which is almost impossible to be determined in patients) or to the relapse behavior of patients for which the TKI treatment has been stopped acting as a surrogate measure of residual leukemic stem cells. A corresponding model analysis studying the molecular relapse risk of patients after TKI stop is currently in progress^32^.

## Discussion

Our statistical analysis of the dynamic (long-term) treatment response of CP-CML patients within the DASISION trial demonstrates differences in the average BCR-ABL dynamics between first-line imatinib and dasatinib treatment. Whereas both TKIs show qualitatively similar response dynamics, we demonstrate that dasatinib leads to a significantly faster molecular response. On the other hand, the detected difference for the average long-term BCR-ABL decline dynamics is small. Although the absolute values for the early BCR-ABL decline are expected to differ quantitatively between the use of ABL and other reference genes (such as GUS)^33,34^, the presented *relative* comparison in the context of a randomized trial does not depend the particular choice of the reference gene.

Our results suggest that although there is also a slight effect on the long-term BCR-ABL decline, the major advantage of dasatinib treatment is evident in the early phase of treatment. Here, the second generation TKI is, on average, more efficient in reducing the tumor load compared to imatinib. Similar results were also obtained from analyzing the primary endpoint of the DASISION trial, which was CCyR after 12 months of therapy^35^: significantly more patients achieved CCyR in the dasatinib arm compared to the imatinib arm (77% vs. 66%, p = 0,007). Additionally, the final analysis with the 5-year follow-up data of the DASISION trial showed a constant efficacy benefit over time for dasatinib^24^. This conforms with our second finding, that once the first phase of the typically bi-phasic BCR-ABL decline has been completed, the average velocity of further tumor load reduction does only marginally differ between the two TKIs. Although dasatinib patients on average more quickly gain deep remissions (such as MR4 or MR4.5), the dynamics of response for treating residual disease differs only slightly between imatinib and dasatinib. Therefore, other criteria, such as side effects, resistance occurrence, or patient compliance should be considered as important criteria for a decision on long-term treatment options.

Beyond the statistical results on the quantitative differences in the treatment effects, we also analyzed the BCR-ABL dynamics in the context of a mechanistic mathematical model. Our simulation results suggest that the above described differences between dasatinib and imatinib treatment are most likely induced by quantitative differences in the TKI effects, especially with regard to the initial treatment efficacy. In particular, the higher rates of gradual TKI-effect onset (r_trans_) in the dasatinib cohort support the notion of a more effective initial therapy.

Our results outline the importance of considering *dynamic* parameters, such as the velocity of BCR-ABL reduction, for predicting the long-term success of the TKI treatment. Whereas response levels at given landmarks (e.g. the BCR-ABL level at three or six months after treatment induction) are clinically relevant for the prediction of treatment failure and the achievement of major/complete cytogenetic or molecular remission, the importance of considering the disease *dynamics* is becoming more and more evident, in particular, as this reflects the underlying mechanisms of disease progression or regression. We and others^7,8,11–14^ promote the idea to also clinically *describe* CML treatment response as a continuous, patient-specific process, and thereby building the foundation of an individualised therapy approach.

This perception is further substantiated by our analyses to quantify the heterogeneity in the response characteristics of different patients, which exceeds the level of drug induced variability (see Fig. 3). Moreover, we could demonstrate that there is no correlation of early and late BCR-ABL dynamics (Fig. 3C) on the level of individual patients. This implies that a prediction about the long-term response of a patient cannot be made based on its initial response alone. In fact, long-term dynamics can only be approximated if at least a part of the secondary, slower slope has been quantified.

By using an established mechanistic model of TKI-treated CML we could demonstrate that optimal parameter configurations for describing the treatment response of individual patients can be identified. Furthermore, by defining confidence regions for the time courses of BCR-ABL levels, we do not only identify an optimal set of parameters, but also a range of scenarios that all adhere with the observed outcome. Using the suggested mapping of measured BCR-ABL values onto *in silico* simulations of the treatment dynamics allows us to provide model-based predictions not only of the expected BCR-ABL values in the peripheral blood, but also for the level of residual leukemic stem cells in the bone marrow. However, our results also indicate that the peripheral blood BCR-ABL levels do not allow to uniquely estimate and, therefore, predict the underlying dynamics at the level of leukemic stem cells. We show that similar response dynamics in the peripheral blood can in principle result from rather different stem cell dynamics. Although this result (partially) relates to the technical problem of incomplete model identifiability, it also points to a more general problem: It theoretically demonstrates that there might be biological processes at the stem cell level that cannot be quantified based on BCR-ABL levels in the peripheral blood alone, thus calling for the identification of additional biomarkers. Nevertheless, because the number of residual leukemic stem cells is most likely the decisive parameter which determines the cure status, our model allows in principle to predict the expected time to “real” cure (i.e. elimination of all leukemic stem cells) on the basis of a (conservative) confidence interval estimation of residual leukemic stem cells. However, in the context of treatment cessation trials it appears that secondary effects like immunological components strongly influence long-term disease control. Including those aspects into a quantitative modelling environment requires both a better understanding of the mechanistic immune interactions as well as their quantification. Corresponding efforts in experimental and conceptual studies are ongoing^36,37^.

As described above, our approach allows for a robust prediction of the long-term molecular response in TKI-treated CML patients. Herein, the quality of the predictions depends considerably on the amount and quality of clinically determined BCR-ABL levels. Further limitations of the approach are related to the appearance of TKI resistance or disease acceleration. Currently, the model assumes constant treatment effects. Along these lines, also effects of non-compliance (i.e., unknown treatment interruptions) are problematic. These are obviously inducing altered treatment effects, which cannot be captured by the model, if time point and durations are not known. Another problem appears if patients respond “too well”, i.e. if BCR-ABL levels quickly approach the PCR quantification limit and stay negative. Although such patients can be considered as good responders, the long-term dynamics can hardly be estimated in these cases. Thus, BCR-ABL negative patients with a persisting BCR-ABL decline (below the quantification limit) cannot be distinguished from those that would show a constant albeit non-detectable residual disease level. While our approach, on the one hand, can partially account for missing information by a compensation from the entire population of similar patients (i.e. mixed-effect modelling), this, on the other hand, bears the danger that the specificity of a particular patient is underestimated.

The availability of high quality time course data of tumor load made CML a primary example for many mathematical modeling approaches. Beyond the statistical description of time courses, several models fostered a strong discussion about underlying mechanisms of disease and treatment progression such as the role of TKI activity in the stem cell compartment^11,38–40^, the role of cellular quiescence^41^, combination therapies^42,43^, resistance occurrence^44^ or the role of the immune system^36,45,46^. All those models are bound to their underlying set of assumptions, which are in many instances motivated by experimental or clinical observations, but need to be understood to appreciate the potential and limitations of each model approach. As for our particular model it is the perception of CML as a competition process of normal and leukemic cells that simplifies a complex set of regulations and escape mechanisms. However, sensitivity analysis and the success of different modeling approaches with slightly diverging assumptions ensures us about the validity of our approach and its generalizability.

Our current work illustrates the role for statistical and dynamical modeling of CML to delineate the potential and the limitations of systems biological approaches for disease management and treatment optimization. While on one side we inherently perceive and describe treatment response as the feature of an individual patient, we, on the other side, use the statistical characterization to adapt a mechanistic model of the underlying treatment. This step allows to derive predictions about unobserved quantities such as the residual leukemic burden in the bone marrow, and thereby builds the basis for patient-specific treatment approaches.

## Electronic supplementary material

Supplementary Material
