# Supplementary material for: Quantitative prediction of long-term molecular response in TKI-treated CML – Lessons from an imatinib versus dasatinib comparison
Source: Sci Rep. 2018 Aug 17;8:12330. doi: 10.1038/s41598-018-29923-4 (PMC6098052; doi:10.1038/s41598-018-29923-4)
Supplement: Supplementary file 1 — Supplementary Material [file 41598_2018_29923_MOESM1_ESM.pdf]

Quantitative prediction of long-term molecular response in TKI-treated CML – Lessons from an imatinib versus dasatinib comparison

Supplementary Methods

Ingmar Glauche, Matthias Kuhn, Christoph Baldow, Philipp Schulze, Tino Rothe, Hendrik Liebscher, Amit Roy, Xiaoning Wang, Ingo Roeder

Statistical modeling

To statistically analyse the relative abundance (in %) of the onco-protein BCR-ABL we describe the BCR-ABL/ABL ratio on the log-scale, i.e. LRATIO = $\log_{10}$(BCR-ABL / ABL * 100). All measurements were further corrected by a factor of 0.81 to ensure transformation of the International Scale. Time courses are described by a logarithmical positive bi-exponential function, i.e.: LRATIO(t) = $\log_{10}[A \cdot \exp(-\alpha t)+B \cdot \exp(-\beta t)]$, with intercept parameters A > B > 0 and slope parameters, α > 0 and $\beta \in \mathbb{R}$. To facilitate these positivity constraints we used a natural logarithm transformation for the parameters A, α and B in the model.

Bi-exponential parameter estimation. We applied a non-linear least-square approach to fit a bi-exponential function LRATIO(t) to the BCR-ABL transcript measurements up to time τ for an individual patient *i*, thereby obtaining a tuple of estimated statistical parameters $\hat{\rho}_i^\tau = (A, \alpha, B, \beta)$ along with an estimated residual variance $\hat{\sigma}_i^2$. The same approach can also be applied to simulated (model-generated) BCR-ABL time courses with model parameters $\vartheta = \left(\mathrm{f}_\omega^{\mathrm{CML}}, \mathrm{f}_\alpha^{\mathrm{CML}}, \mathrm{r}_{\mathrm{deg}}, \mathrm{LRATIO}_{\mathrm{init}}, \mathrm{r}_{\mathrm{trans}}\right)$, thereby obtaining $\hat{\rho}_\vartheta^\tau$.
The approach accounts for left-censored observations, i.e. (BCR-ABL-negative) measurements that are below the quantification limit (QL) of the RT-PCR. For each RT-PCR measurement we obtain an individual estimate of its QL, based on the available ABL reads (QL = 3/ABL * 100) (Cross et al., 2015). Different methods for handling censored observations are discussed in Beal, 2001. In order to obtain conservative estimates for the time courses, we implemented a related adaptive replacement approach: we replaced censored observations with their QL value when the fitted value was above QL but considered a censored observation to match the fit when the fit itself was below QL as well (i.e. model predictions below the QL are not penalized). As up to 80% of observations per patient are below QL we generally weighted below QL observations with factor ½ in order to get robust patient fits. For bi-exponential fits of simulated BCR-ABL/ABL time courses, we weighted observations above 1 % and below 0.001 % (MR5) with factor ½ to reduce the influence of stochastic effects and to minimize their impact on the final fits.

Confidence intervals. We used parametric bootstrap to obtain confidence intervals for the mean LRATIO value for a patient *i* at any given time point. For a given data set, we started from the predicted LRATIO at the observed time points and repeatedly added a random measurement error sampled from a normal distribution with mean 0 and with variance $\hat{\sigma}_i^2$, the estimated residual variance from the bi-exponential model fit. We also applied the observed lower quantification limits at the affected time points. We repeatedly fit the model to the simulated response data and extracted the range of 95% of the predicted mean LRATIO values, thereby defining the confidence interval.

Group Comparison of imatinib versus dasatinib. For the assessment of treatment differences between imatinib and dasatinib therapy with respect to the LRATIO levels in the peripheral blood, we applied a non-linear mixed effect (NLME) model using maximum likelihood (ML) estimation with the stochastic approximation EM algorithm as implemented in Monolix.
Model selection was applied as follows: We started with a bi-exponential model that incorporated solely patient-level random effects for the parameters α, B and β. We did not include a patient-level random effect for the first intercept A as this parameter showed negligible patient to patient variability. Further model selection, i.e. the decision which treatment effects to include in the final

NLME-model, was guided by Akaike's information criterion (AIC). The finally selected model contains random effects for all parameters but initial slope parameter A, while fixed treatment group effects for initial slope α and second slope β. We applied Wald tests to assess the statistical significance of fixed-effect group effects. Residual plots and the distribution of the predicted random effects of the final NLME-model did not reveal any evident violations of the assumptions of NLME-models. The model fit was robust with respect to the choice of initial start values for the parameters. Our retained model is summarized in Suppl. Table S1.

Coefficient	***Estimate***	***Std.Error***	***p-value***
ln(A)	3.62	0.044	
ln(α) imatinib	-0.395	0.050	
ln(α) change dasatinib	0.55	0.069	<0.0001
ln(B)	-1.63	0.103	
β imatinib	0.0392	0.003	
β change dasatinib	0.009	0.004	0.0323
$\omega_{ln(\alpha)}$	0.575	0.0292	
$\omega_{ln(B)}$	1.88	0.081	
ω_{β}	0.0398	0.002	
residual std. deviation σ	0.405	0.004	

Suppl. Table S1: Fixed effect coefficients and variance components as standard deviations of the finally selected non-linear mixed effect model with Wald significance tests for fixed-effect group effects. The parameters A, α and B are reported on log-scale. (AIC: 8358.07)

Furthermore, as it is known that measurements of high tumor load (i.e. BCR-ABL ratio > 10%) are more uncertain due to the usage of the ABL1-reference gene, we assessed the impact of these high tumor load measurements on our results and performed the identical analysis when BCR-ABL values > 10 % are either given half weight (Suppl. Table S2) or are dropped (Suppl. Table S3) from the dataset. The treatment effect estimates are consistent and also the statistical significance persists in both scenarios.

Coefficient	***Estimate***	***Std.Error***	***p-value***
ln(A)	3.45	0.057	
ln(α) imatinib	-0.439	0.052	
ln(α) change dasatinib	0.55	0.071	<0.0001
ln(B)	-1.65	0.102	
β imatinib	0.0396	0.003	
β change dasatinib	0.0094	0.004	0.0139
$\omega_{ln(\alpha)}$	0.583	0.0352	
$\omega_{ln(B)}$	1.84	0.0813	
ω_{β}	0.0353	0.0018	
residual std. deviation σ	0.396	0.0048	

Suppl. Table S2: Fixed effect coefficients and variance components as standard deviations of the non-linear mixed effect model when all values above 10 % are given half weight. Wald significance tests for fixed-effect group effects are given. The parameters A, α and B are reported on log-scale. (AIC: 8372.7)

Coefficient	*Estimate*	*Std.Error*	*p-value*
ln(A)	2.17	0.0914	
ln(α) imatinib	-0.861	0.071	
ln(α) change dasatinib	0.583	0.0786	<0.0001
ln(B)	-1.84	0.101	
β imatinib	0.0381	0.0025	
β change dasatinib	0.0118	0.0032	0.0002
$\omega_{ln(\alpha)}$	0.636	0.0353	
$\omega_{ln(B)}$	1.77	0.0805	
ω_{β}	0.0288	0.0016	
residual std. deviation σ	0.394	0.0048	

Suppl. Table S3: Fixed effect coefficients and variance components as standard deviations of the non-linear mixed effect model neglecting all values above 10 %. Wald significance tests for fixed-effect group effects are given. The parameters A, α and B are reported on log-scale. (AIC: 7146.77 on smaller data set)

Time to event data. To analyze time-to-event data, such as time to progression or time to major molecular response (MMR, i.e. BCR-ABL below 0.1%), we used cumulative incidence estimates with death as a competing risk event. We defined high and low risk subgroups based on cut-off values of halfing time, initial slope α or long-term slope β, respectively. The cutoff values were defined by maximizing the average of sensitivity and specificity (Youden-index) on the data. We used the Gray-test to test for significant differences of the cumulative incidence functions between the high and low risk group (R-package cmprsk).

Simulation algorithm & model description

The described model of HSC organization and leukemia is implemented as a single-cell based, stochastic process. We used a discrete time step approach, with $\Delta t = 1$ hour, where every single cell is updated according to defined rules including stochastic decisions.

The status and state of a model cell, irrespective of its type, is given by its affinity a, its membership to a signaling context $m \in \{A, \Omega\}$ and its position in the cell cycle c. The cell cycle for an activated cell in the signaling context Ω is modeled as a sequence of $G1$ phase, S phase, and $G2/M$ phase, in which the duration of latter phases (τ_S and $\tau_{G2/M}$) is fixed. To realize an update step, the actual total number of cells with $a > a_{\min}$ in signaling context A ($N_A(t)$) and Ω ($N_\Omega(t)$) is determined. Based on these numbers and according to its actual signaling context, the status of each model stem cell is updated as follows:

($\mathbf{A}$) The cell changes to signaling context Ω or stays in A with probabilities ω and $1 - \omega$, respectively. If it stays in A, its cell specific affinity a is increased by the factor r (regereneration coefficient) up to $a_{\max} = 1$. I the cell changes to signaling context Ω, its position in the cell cycle will be set to the beginning of S-Phase.

($\mathbf{\Omega}$) The cell changes to signaling context A or stays in Ω with probabilities α and $1 - \alpha$, respectively. Herein, a change to signaling context A is only possible if the cell is in $G1$-Phase and a > $a_{\min}$, ending up in $G0$-Phase (cell inactivity). If a cell stays in signaling context Ω, the affinity a is decreased by the division of the differentiation coefficient d and the cell cycle counter is incremented. In case the cell cycle is completed, i.e. $c(t) = \tau_{G_1} + \tau_S + \tau_{G2/M}$), $c(t)$ is set to 0 and a new identical cell is generated (cell division). In case $a < a_{\min}$ cells can not reenter into the signaling context A and continue development in Ω (differentiation/maturation).

All cells with $a > a_{\min}$ are denoted as stem cells, whereas cells with $a \leq a_{\min}$ corresponds to differentiating cells. The differentiating cells undergo an proliferative phase of 480 hours before they mature for another 192 hours. BCR-ABL/ABL ratios are calculated based on the fraction of leukemic cells among this pool of maturing cells.
The transition probabilities α and ω depend on the actual affinity $a(t)$ of the cell, on the fixed parameters $a_{\min}$ and $a_{\max}$ and on the transition characteristics f_α and f_ω, respectively:

$$\alpha = \frac{a(t)}{a_{\max}} f_\alpha(N_A(t))$$

$$\omega = \frac{a_{\min}}{a(t)} f_\omega(N_\Omega(t))$$

The transition characteristics f_α and f_ω are crucial regulators for the description of the dynamic differences between healthy and leukaemic cells as well as the effect of tyrosine kinase inhibitors. Besides their cell type it depends also on the cell numbers of the target context. They are modelled by a general class of sigmoid functions:

$$f_{\alpha/\omega}(N_{A/\Omega}) = \frac{1}{\nu_1 + \nu_2 \cdot \exp\left(\nu_3 \cdot \frac{N_{A/\Omega}}{\tilde{N}_{A/\Omega}}\right)} + \nu_4$$

The parameters ν_1, ν_2, ν_3 and ν_4 determine the shape of the transition functions $f_{\alpha/\omega}$. $\tilde{N}_{A/\Omega}$ is a scaling factor of the corresponding compartment. ν_1, ν_2, ν_3 and ν_4 can be uniquely determined by more intuitive values $f_{\alpha/\omega}(0)$, $f_{\alpha/\omega}(\frac{\tilde{N}_{A/\Omega}}{2})$, $f_{\alpha/\omega}(\tilde{N}_{A/\Omega})$ and $f_{\alpha/\omega}(\infty) := \lim_{N_{A/\Omega}\to\infty} f_{\alpha/\omega}(N_{A/\Omega})$:

$$\nu_1 = \frac{h_1 h_3 - h_2^2}{h_1 + h_3 - 2h_2}$$

$$\nu_2 = h_1 - \nu_1$$

$$\nu_3 = \ln\left(\frac{h_3 - \nu_1}{\nu_2}\right)$$

$$\nu_4 = f_{\alpha/\omega}(\infty)$$

with

$$h_1 = (f_{\alpha/\omega}(0) - f_{\alpha/\omega}(\infty))^{-1}$$

$$h_2 = (f_{\alpha/\omega}\left(\frac{\tilde{N}_{A/\Omega}}{2}\right) - f_{\alpha/\omega}(\infty))^{-1}$$

$$h_3 = (f_{\alpha/\omega}(\tilde{N}_{A/\Omega}) - f_{\alpha/\omega}(\infty))^{-1}$$

In order to explain the competitive advantage of leukemic compared with healthy cells, we assume that the leukemic cells have an increased and unregulated proliferative activity. Technically, the transition characteristics f_A and f_Ω, which describe the transit of cells between A and Ω, differ such that leukaemic cells have (I) an increased and cell number-independent probability per time step to be activated into cycle and (II) a slightly increased propensity to find an empty niche site, which is modelled by an increased probability to change to the niche- like signalling context A. Both assumptions are necessary to consistently explain characteristics of CML pathogenesis (Horn, Loeffler, & Roeder, 2008; Roeder et al., 2006). For a fully developed CML, this leads to a higher absolute number of leukemic cells compare to normal cells in the homeostatic situation. However, as the regulation of the normal cells is unaltered, those cells are limited in their proliferative activity and are therefore slowly outcompeted. This is an indirect effect caused by different regulating mechanism rather than a direct spatial competition. A conceptual sketch of the model is provided in Figure 2B of the main document.

Due to performance reasons, we precomputed simulations in certain parameter ranges, based on the results of previous studies. Technically, we varied the degradation rate r_{deg}, the rate of gradual TKI onset r_{trans}, the activation function f_ω^{CML} for leukemic cells, the deactivation function f_α^{CML} for leukemic cells, and the initial leukemic tumour load $LRATIO_{init}$ (see Suppl. Table S4). The

simulation of all possible combinations lead to a parameter grid of 270,400 combinations $\vartheta = (f_{\omega}^{CML}, f_{\alpha}^{CML}, r_{deg}, LRATIO_{init}, r_{trans})$, building the base of a look-up table. Given the full combinatorics and performing five simulation runs per design point to obtain a robust estimate of a typical BCR-ABL time course (which is a stochastic process) over 8 years of follow-up, we ended up with a total of 1,352,000 simulation runs.

Parameter	***min***	***max***	***step***	***#***
r_{deg}	1.5	7.5	0.4	16
r_{trans}	2	6.8	0.4	13
f_{ω}^{CML}	0.01	0.1	0.01	10
f_{α}^{CML}	0.5	0.98	0.04	13
$LRATIO_{init}$	10	99	10	10

Suppl. Table S4: Parameter ranges as the basis of the lookup table. All possible combinations were presimulated in order to use efficiently for comparison with patients' data.

References

Cross, N. C., White, H. E., Colomer, D., Ehrencrona, H., Foroni, L., Gottardi, E., . . . Hochhaus, A. (2015). Laboratory recommendations for scoring deep molecular responses following treatment for chronic myeloid leukemia. *Leukemia, 29*(5), 999-1003. doi:10.1038/leu.2015.29

Beal, S. L. (2001). Ways to fit a PK model with some data below the quantification limit. *J Pharmacokinet Pharmacodyn, 28*(5), 481-504.

Horn, M., Loeffler, M., & Roeder, I. (2008). Mathematical modeling of genesis and treatment of chronic myeloid leukemia. *Cells Tissues Organs, 188*(1-2), 236-247. doi:10.1159/000118786

Roeder, I., Horn, M., Glauche, I., Hochhaus, A., Mueller, M. C., & Loeffler, M. (2006). Dynamic modeling of imatinib-treated chronic myeloid leukemia: functional insights and clinical implications. *Nat Med, 12*(10), 1181-1184. doi:10.1038/nm1487

Figure S1

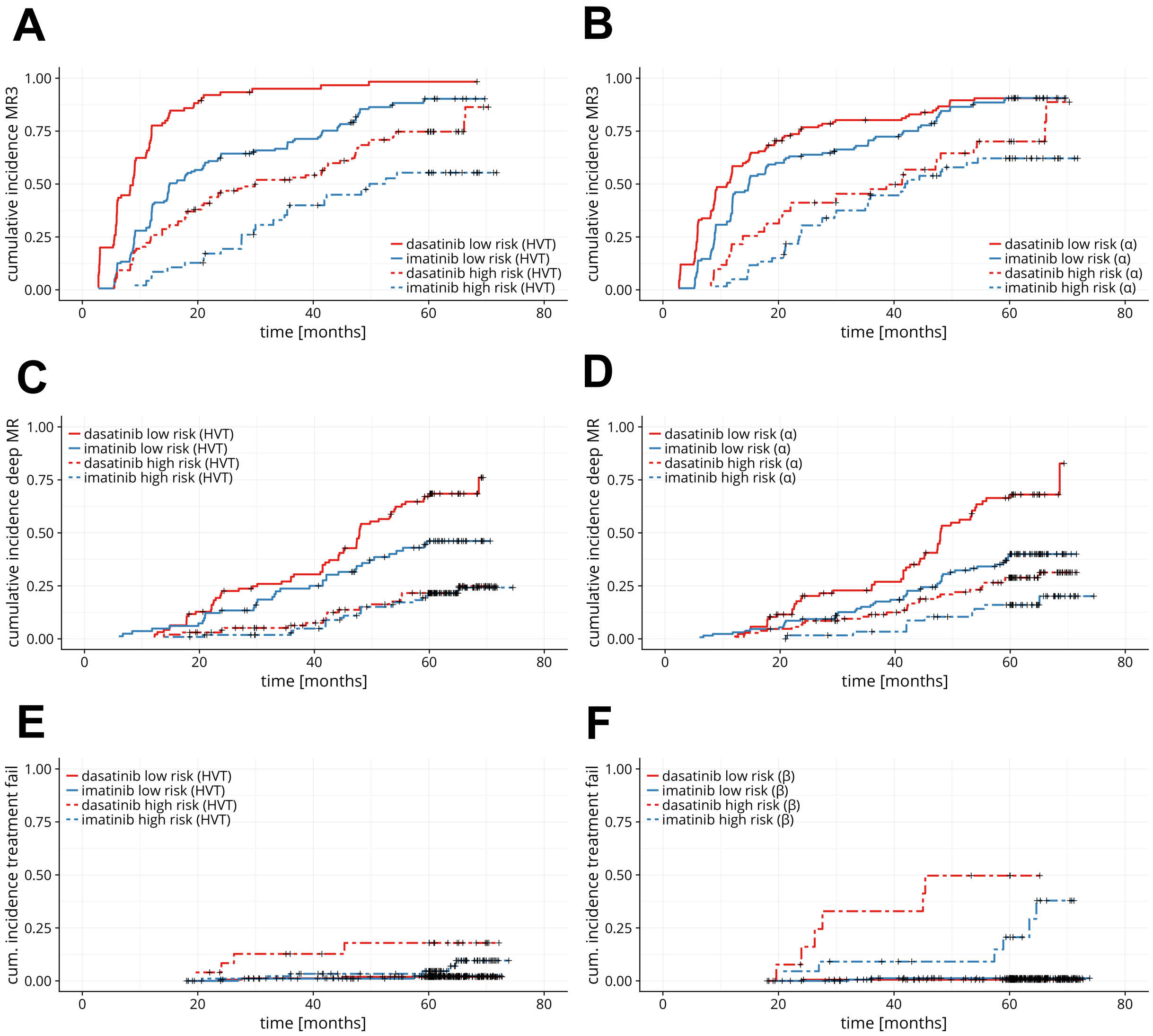

Supplementary Figure S1. Predictability of slope analysis for clinical outcomes. Cohorts are separated into a high and low risk group by maximizing the sum of sensitivity and specificity (ROC analysis) with respect to the intended outcome for dasatinib and imatinib individually. The determined cut points together with the results (i.e., p-values) of testing for equality of the cumulative incidence in high and low risk group are given for each treatment arm, respectively. (**A,B)** Halving time and α-slope as predictors for achievement of major molecular remission (MMR, BCR-ABL/ABL < 0.1%). Threshold value for HVT (subfigure A): dasatinib HVT_{crit} = 0.508 (p=4.4e-08) and imatinib HVT_{crit} = 1.433 (p=9.2e-07). Threshold value for α-slope (subfigure B): dasatinib α_{crit} = -0.808 (p = 8.4e-05) and imatinib α_{crit} = -0.554 (p=2.4e-06). **(C,D)** Halving time and α-slope as predictors for achievement of deep molecular remission (deepMR, BCR-ABL not detectable). Threshold value for HVT (subfigure C): dasatinib HVT_{crit} = 0.548 (p=3.9e-11) and imatinib HVT_{crit} = 0.754 (p=3.3e-4). Threshold value for α-slope (subfigure D): dasatinib α_{crit} = -1.52 (p=5.6e-06) and imatinib α_{crit} = -0.566 (p=3.8e-3). **(E,F)** Halving time and secondary BCR-ABL decline (β-slope) as predictors for progression free survival. Threshold value for HVT (subfigure E): dasatinib HVT_{crit} = 1.25 (p=3.9e-4) and imatinib HVT_{crit} = 0.825 (p=0.14). Threshold value for β-slope (subfigure F): dasatinib β_{crit} = 0.053 (p=3.3e-16) and imatinib β_{crit} = 0.037 (p=1.2e-8).

Figure S2

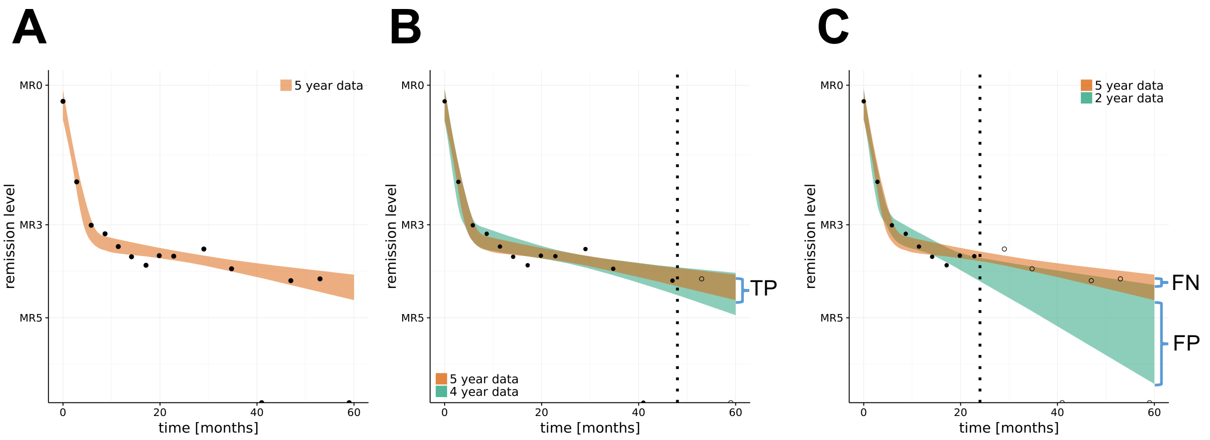

Supplementary Figure S2. Accuracy of model predictions. (A) Illustration of the confidence region (red shaded) of the mean BCR-ABL level for a given patient (87) as a function of treatment time. **(B)** Corresponding confidence region if only data until year 4 of treatment is available (green shaded). The overlapping region is indicated as true positive (TP) prediction. **(C)** Corresponding confidence region if only data until year 2 of treatment is available (green shaded). The non-overlapping regions are marked as false positives (FP) and false negatives (FN).

Figure S3

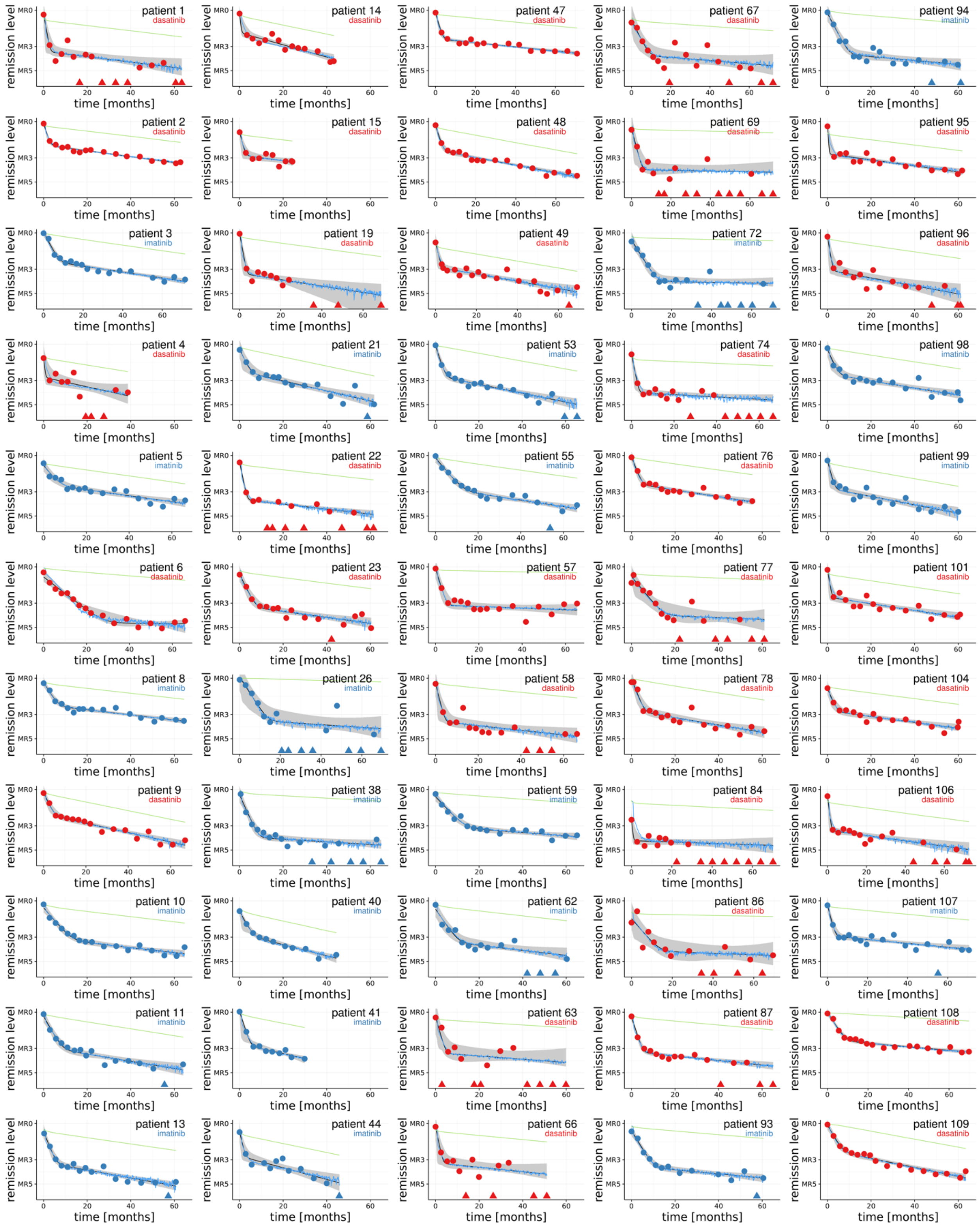
patient 1
dasatinib
patient 14
dasatinib
patient 47
dasatinib
patient 67
dasatinib
patient 94
imatinib
patient 2
dasatinib
patient 15
dasatinib
patient 48
dasatinib
patient 69
dasatinib
patient 95
dasatinib
patient 3
imatinib
patient 19
dasatinib
patient 49
dasatinib
patient 72
imatinib
patient 96
dasatinib
patient 4
dasatinib
patient 21
imatinib
patient 53
imatinib
patient 74
dasatinib
patient 98
imatinib
patient 5
imatinib
patient 22
dasatinib
patient 55
imatinib
patient 76
dasatinib
patient 99
imatinib
patient 6
dasatinib
patient 23
dasatinib
patient 57
dasatinib
patient 77
dasatinib
patient 101
dasatinib
patient 8
imatinib
patient 26
imatinib
patient 58
dasatinib
patient 78
dasatinib
patient 104
dasatinib
patient 9
dasatinib
patient 38
imatinib
patient 59
imatinib
patient 84
dasatinib
patient 106
dasatinib
patient 10
imatinib
patient 40
imatinib
patient 62
imatinib
patient 86
dasatinib
patient 107
imatinib
patient 11
imatinib
patient 41
imatinib
patient 63
dasatinib
patient 87
dasatinib
patient 108
dasatinib
patient 13
imatinib
patient 44
imatinib
patient 66
dasatinib
patient 93
imatinib
patient 109
dasatinib
remission level
MR0
MR3
MR5
time [months]
0
20
40
60

Figure S3 extended

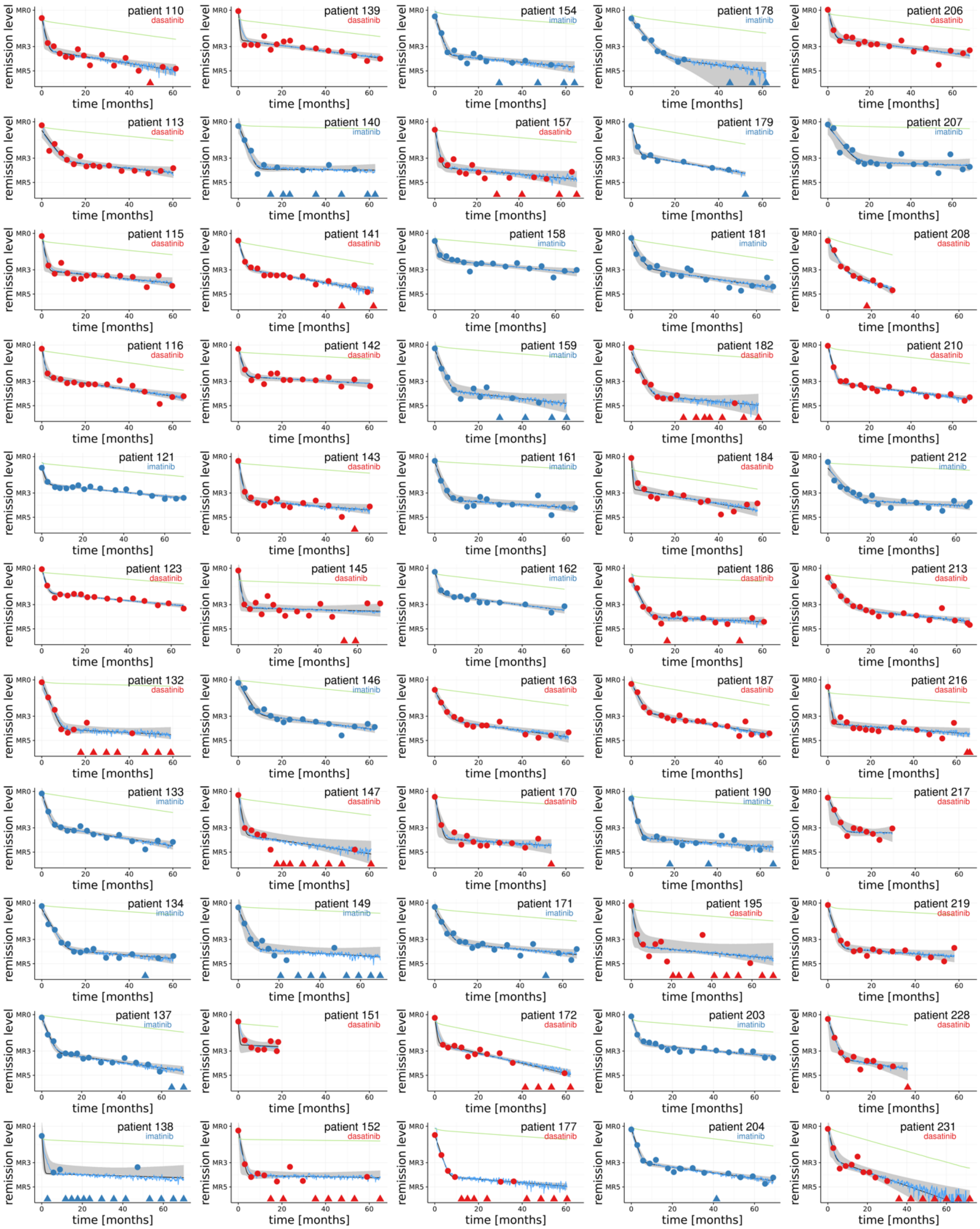

Figure S3 extended

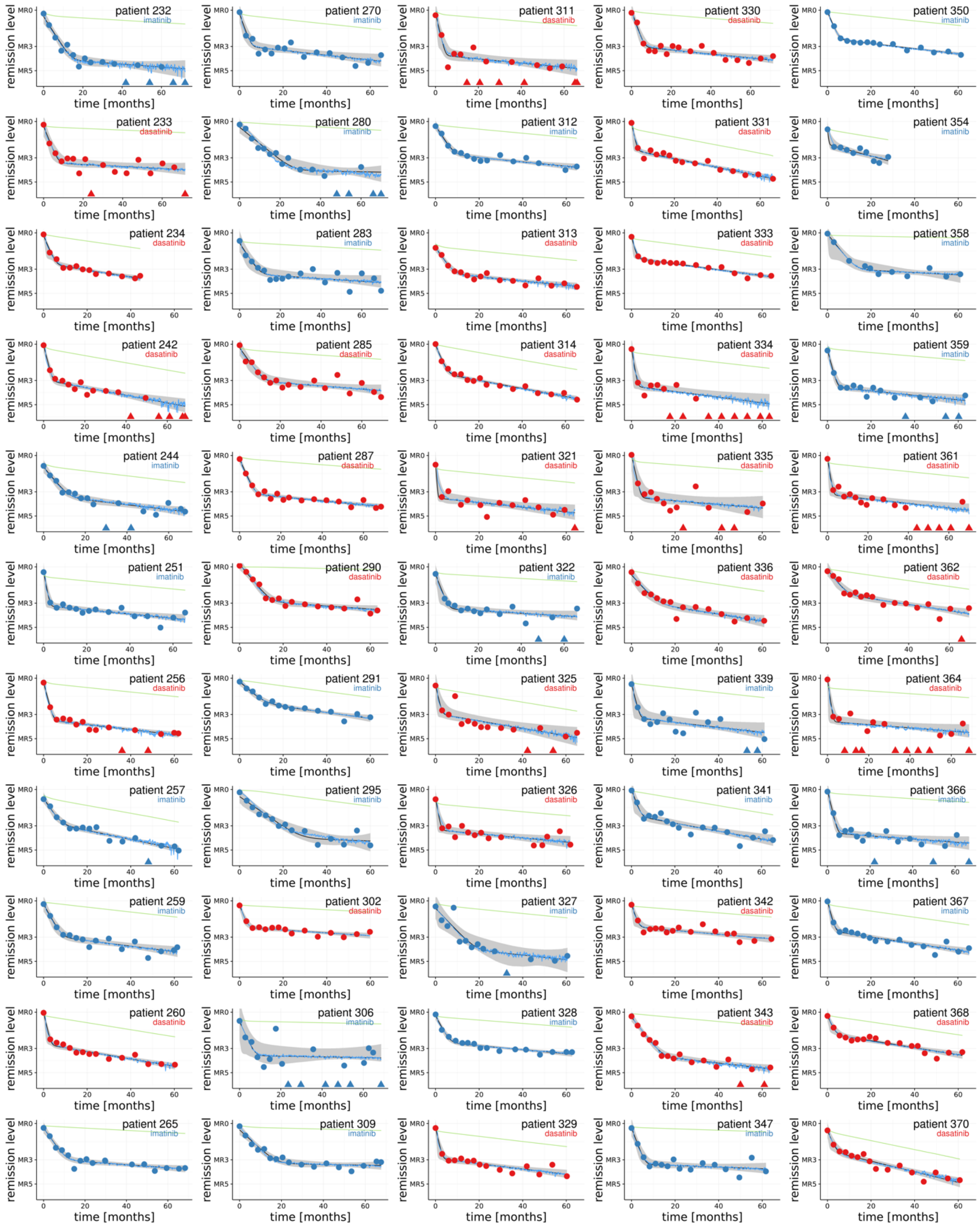

Figure S3 extended

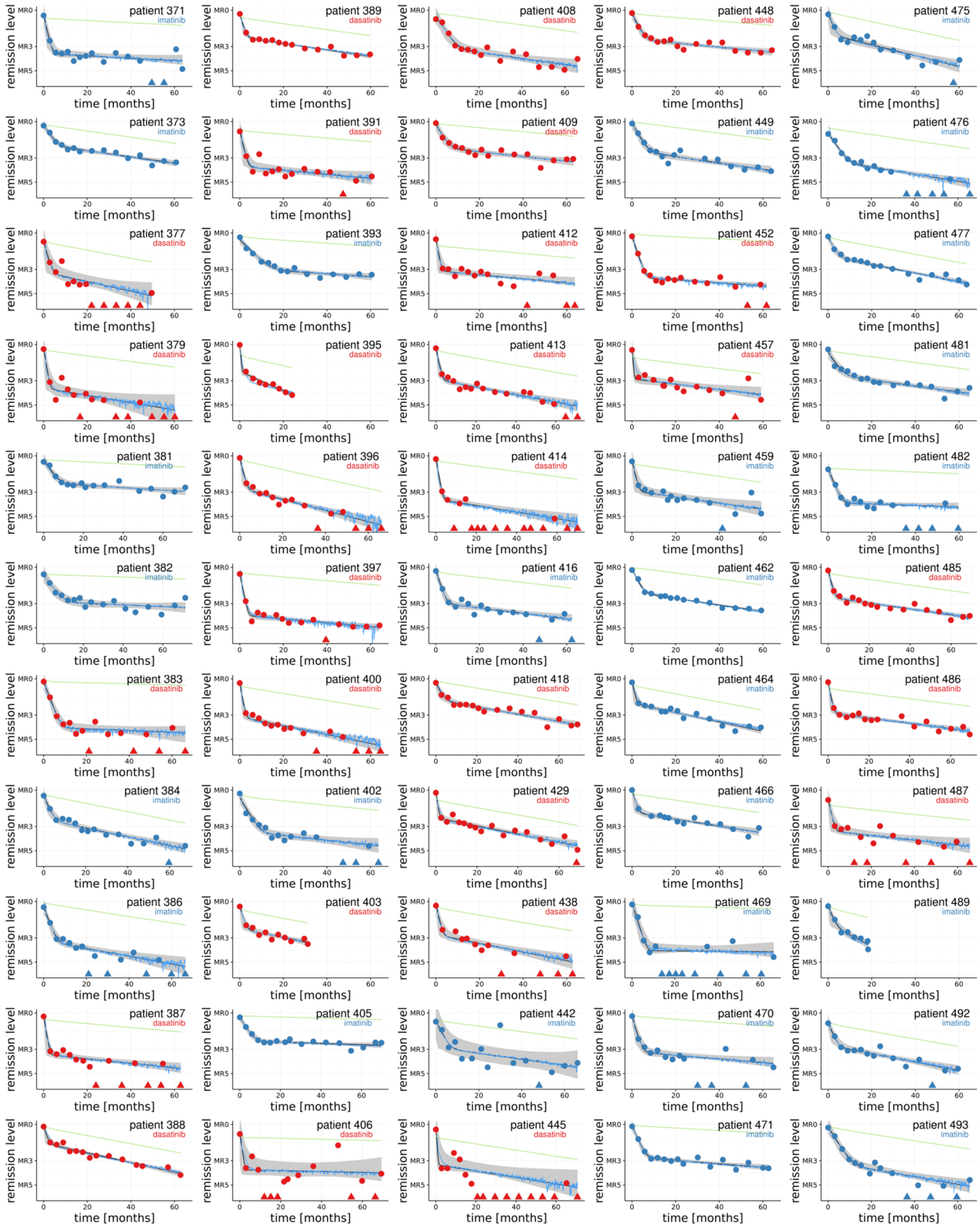

Figure S3 extended

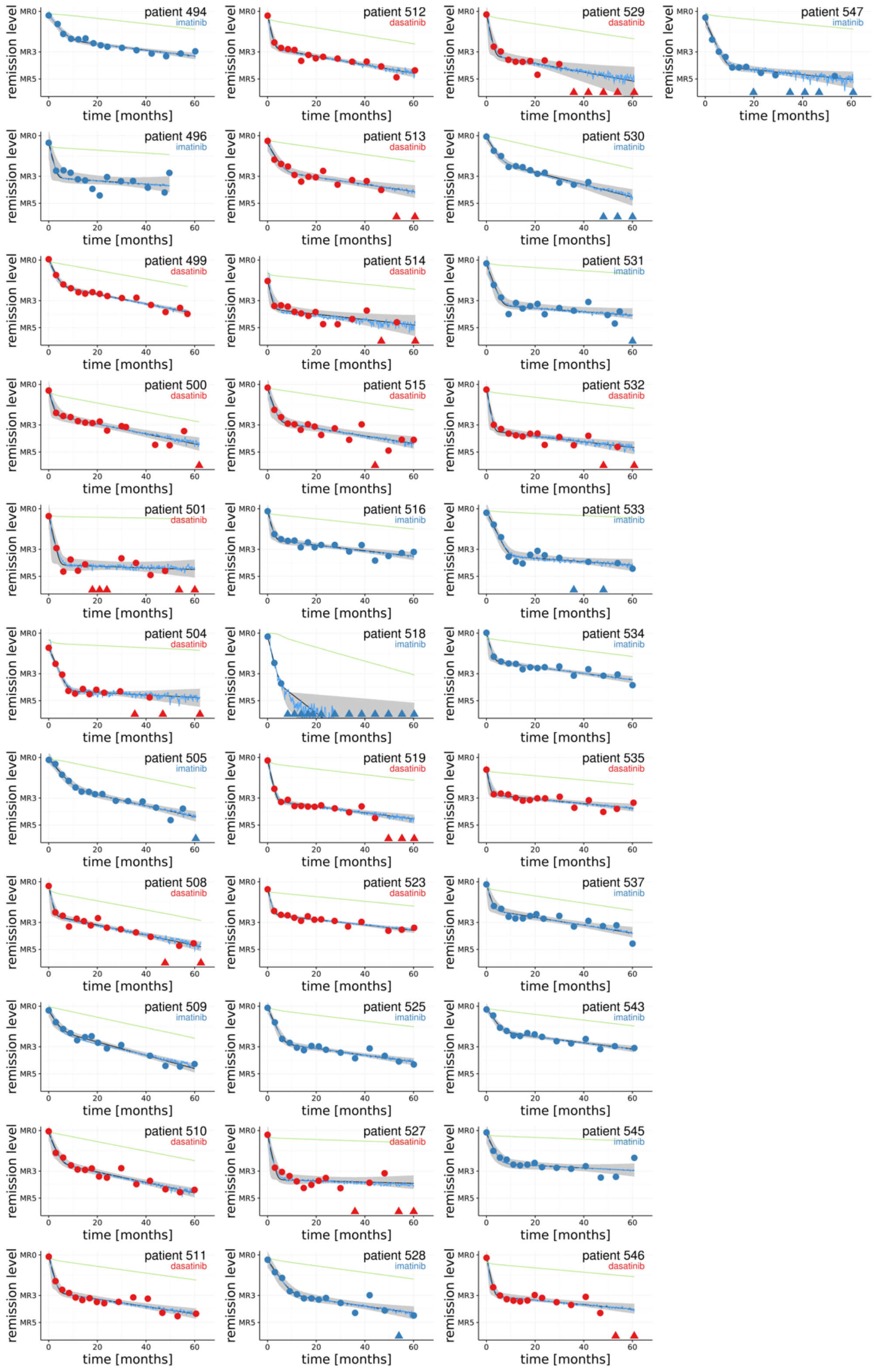

Supplementary Figure S3. Time courses of all 254 that pass the model filter. Each time course is supplemented with the statistical bi-exponential fit $\hat{\rho}_i^\tau$ (black), its confidence region obtained from the parametric bootstrap method (grey) and the most suitable model simulation $\hat{\vartheta}_i^\tau$(blue for peripheral blood and green for LSCs). Triangles indicate non-detectable values.

Figure S4

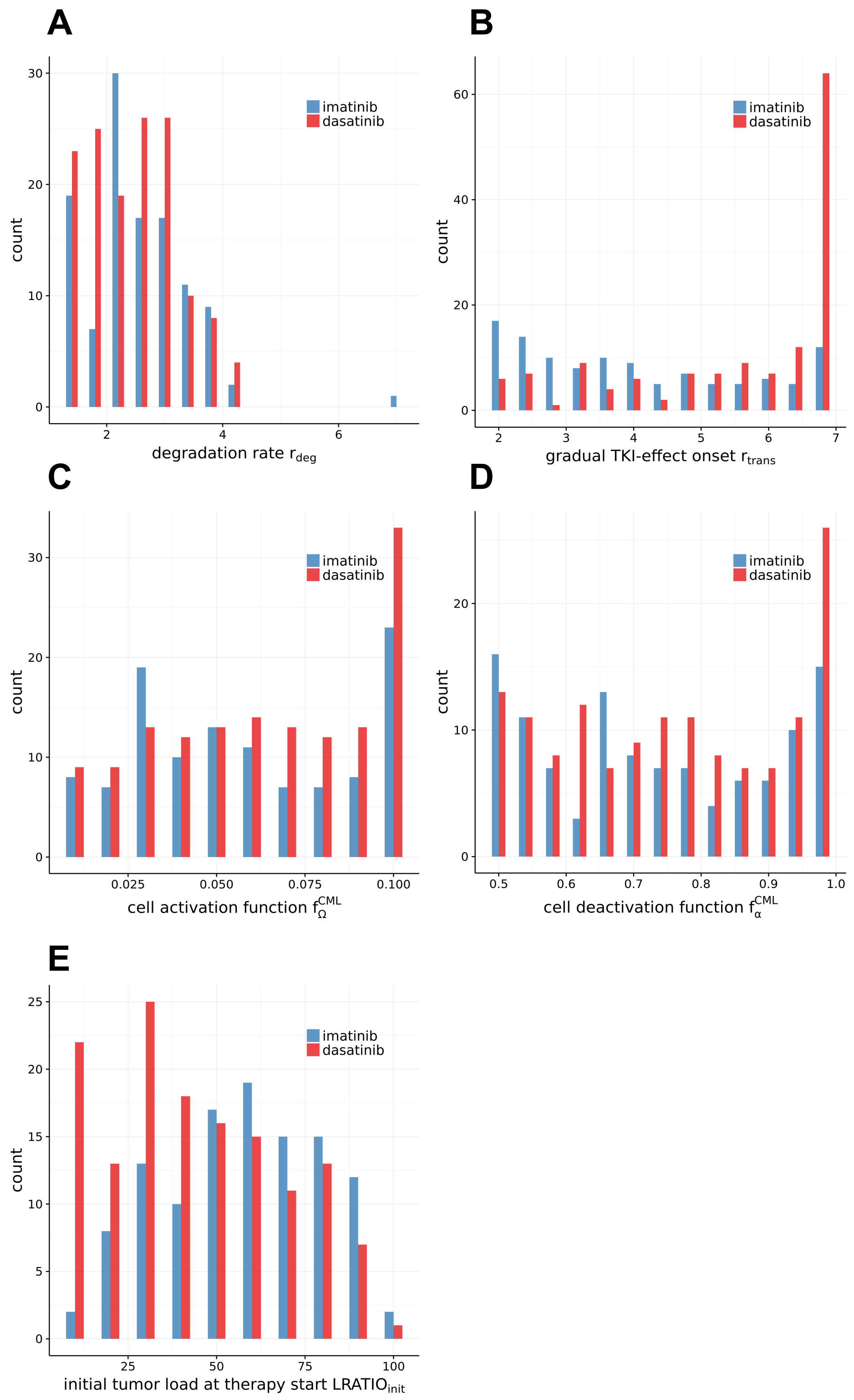

Supplementary Figure S4. Comparison of dynamic modelling parameters. Subfigures show histograms of **(A)** the patient specific degradation rate r_{deg} , **(B)** the rate of gradual TKI onset r_{trans} , **(C)** the reduced activation function (f_{ω}^{CML}), **(D)** the rate by which leukemic stem cells re-enter into quiescence (f_{α}^{CML}) and **(E)** the initial tumor load at therapy start $LRATIO_{init}$, and obtained for the most optimal parameter setting Q^*_{min} for each patient.

Figure S5

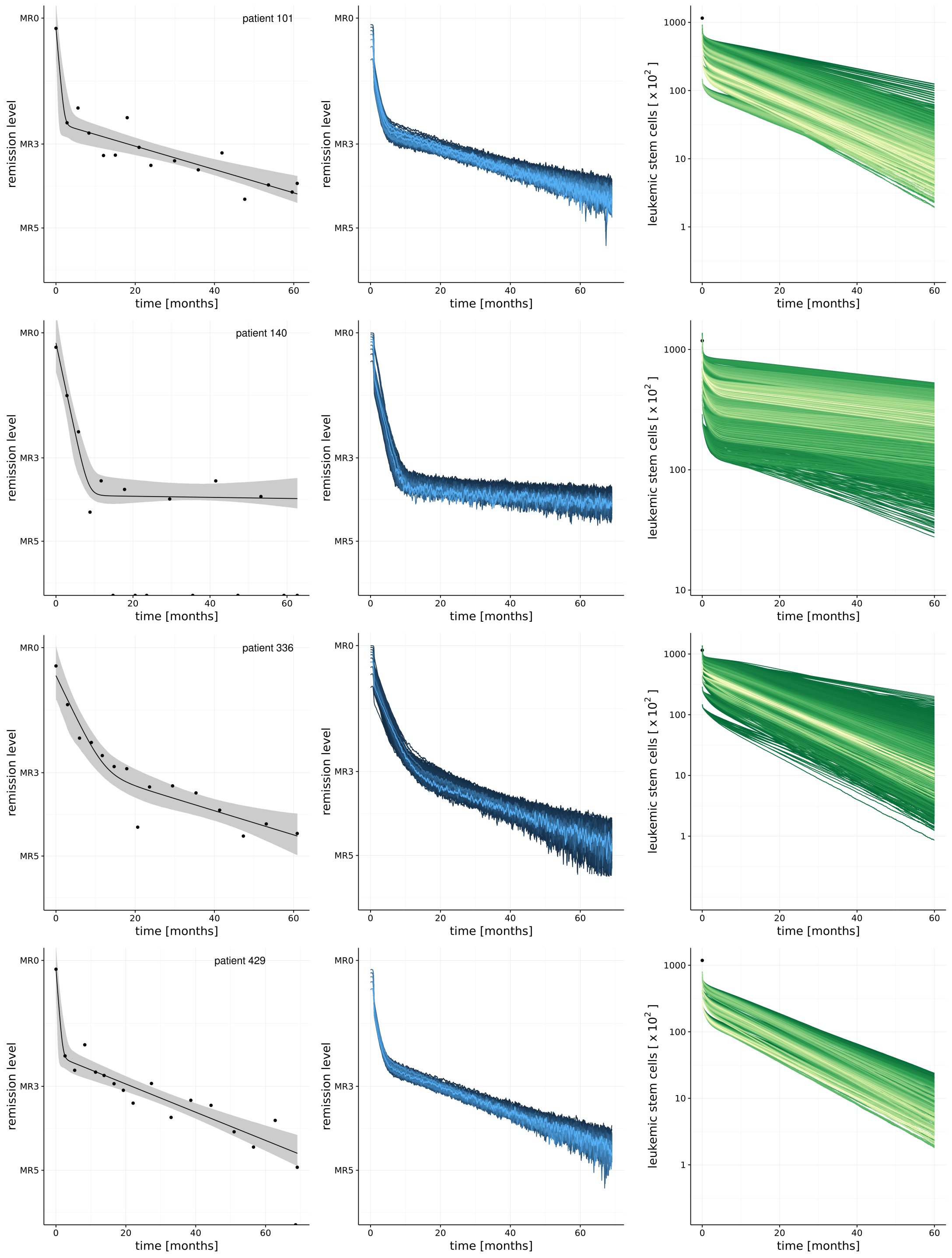

Supplementary Figure S5. Illustration of the model fitting routine for different patients. Left panel indicates the optimal biexponential fit $\hat{\rho}_i^{\tau}$ with the resulting confidence region. The middle panel shows the corresponding set of model simulations Θ_i^{τ} for the particular patient in the peripheral blood. The right panel indicates the predictions for the residual leukemic stem cells. Brighter colors (both blue and green) indicate model approximations with closer similarity to the patient's biexponential fit $\hat{\rho}_i^{\tau}$.

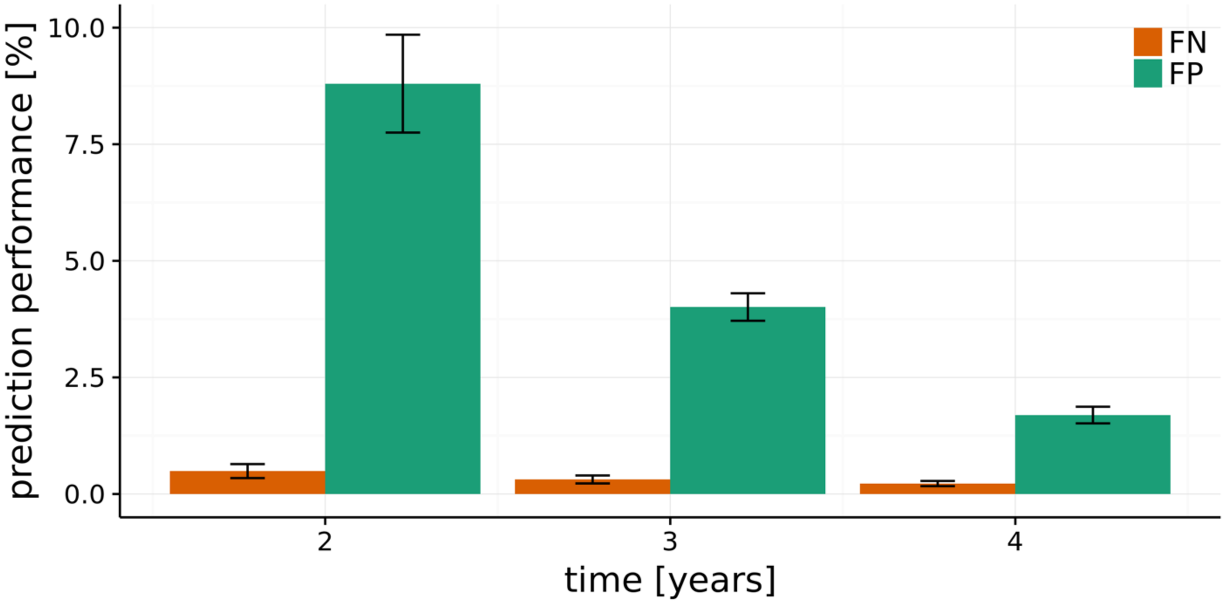

Supplementary Figure S6. Prediction accuracy of estimated model parameters within the patient's confidence region down-weighting values with BCR-ABL ratio > 10% by ½. False positives (FP) and false negatives (FN) rates for predictions of 5 year outcomes are given as a function of the available (shorter) observation period (n = 214).

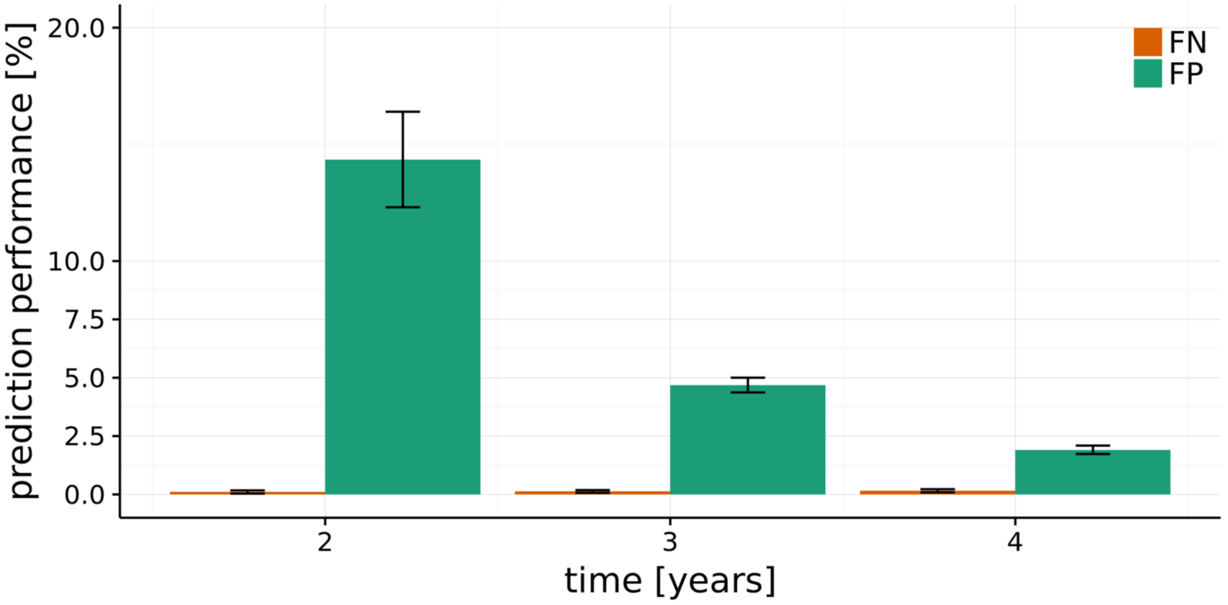

Supplementary Figure S7. Prediction accuracy of estimated model parameters within the patient's confidence region omitting values with BCR-ABL ratio > 10%. False positives (FP) and false negatives (FN) rates for predictions of 5 year outcomes are given as a function of the available (shorter) observation period (n = 211). The slightly higher FP rate (compared to Fig. 4E and S6) results from the higher uncertainty (due to fewer measurements), while the overall trend of increasingly precise predictions with longer observation times remains unchanged.
